# Antimalarial drug targets in Plasmodium falciparum predicted by stage-specific metabolic network analysis

**DOI:** 10.1186/1752-0509-4-120

**Published:** 2010-08-31

**Authors:** Carola Huthmacher, Andreas Hoppe, Sascha Bulik, Hermann-Georg Holzhütter

**Affiliations:** 1 Institute of Biochemistry, Charité, Monbijoustraße 2, 10117 Berlin, Germany

## Abstract

**Background:**

Despite enormous efforts to combat malaria the disease still afflicts up to half a billion people each year of which more than one million die. Currently no approved vaccine is available and resistances to antimalarials are widely spread. Hence, new antimalarial drugs are urgently needed.

**Results:**

Here, we present a computational analysis of the metabolism of *Plasmodium falciparum*, the deadliest malaria pathogen. We assembled a compartmentalized metabolic model and predicted life cycle stage specific metabolism with the help of a flux balance approach that integrates gene expression data. Predicted metabolite exchanges between parasite and host were found to be in good accordance with experimental findings when the parasite's metabolic network was embedded into that of its host (erythrocyte). Knock-out simulations identified 307 indispensable metabolic reactions within the parasite. 35 out of 57 experimentally demonstrated essential enzymes were recovered and another 16 enzymes, if additionally the assumption was made that nutrient uptake from the host cell is limited and all reactions catalyzed by the inhibited enzyme are blocked. This predicted set of putative drug targets, shown to be enriched with true targets by a factor of at least 2.75, was further analyzed with respect to homology to human enzymes, functional similarity to therapeutic targets in other organisms and their predicted potency for prophylaxis and disease treatment.

**Conclusions:**

The results suggest that the set of essential enzymes predicted by our flux balance approach represents a promising starting point for further drug development.

## Background

Malaria represents one of the major health issues worldwide. According to WHO estimations there were around 250 million clinical cases and about 1 million disease related deaths in 2006 [1]. About 50% of the world population lives in endemic areas, situated mostly in Africa, Asia, and South-America [1].

Especially in Africa, where about 90% of the malaria cases occur [1], the disease has significant negative impact on the economic development [2]. Spreading resistances have been developed over time to all but one of the five major classes of antimalarial drugs [3]. As up to now no approved vaccine is available, new effective and affordable drugs are urgently needed.

Malaria is caused by protozoan parasites of the genus Plasmodium, of which *Plasmodium falciparum *is responsible for 90% of disease related deaths [1]. The life cycle of this unicellular eukaryote is quite complex, since it involves different hosts and tissues [4]. Parasites injected via a mosquito bite into the human blood stream first replicate within the liver and subsequently periodically within erythrocytes. Mosquitos taking a blood meal on infected individuals ingest parasites into the midgut, where the only phase of sexual replication takes place. Subsequently, the parasites migrate to the mosquito's salivary gland, in order to be reinjected into the human host. Multiple genome-scale gene expression analyses have been conducted in the last years that allow for an insight into the changes of the transcriptome of *P. falciparum *over the course of its development. We evaluated *in vitro *and *in vivo *expression data from five studies [5-9] that cover multiple time points during liver, blood, and mosquito stage of the parasite. These data sets provide a starting point for further analyses of metabolic peculiarities during different time points of parasite development.

Flux balance analysis (FBA) [10] is a computational method to gain insight into the metabolic behavior and capabilities of a cell. In contrast to kinetic modeling, no detailed rate equations are required, predestining the method for large metabolic networks which typically lack full kinetic characterization of all enzymes. FBA only necessitates knowledge about the stoichiometry of a metabolic network as well as the metabolic target functions underlying the cell of interest (e.g., biomass production). Prediction quality improves if in addition the present metabolic environment is known. Based on this information the outcome of perturbations imposed on a cellular system (e.g., variation in available nutrients or the inhibition of certain enzymes) can be predicted as has been shown for a variety of organisms [11-22]. FBA facilitates the prediction of reactions essential for the production of important metabolites, thus narrows down the drug target search space and ultimately lowers the cost for the whole drug design process. To our knowledge, network based analyses of the metabolism of *P. falciparum *have so far only been applied by Yeh *et al. *[23] and Fatumo *et al. *[24]. Both authors performed *in silico *knock-out studies to uncover putative drug targets in the parasite's metabolism. However, both publications studied metabolic networks derived from the BioCyc database that were not processed further using additional information such as cellular compartments and transport processes and therefore are of limited usefulness. Moreover, they did not consider the different life cycle stages of the parasite, during which the metabolism might vary due to varying availability of nutrients in the different cellular environments.

The goal of this study was to explore stage-specific metabolism of *P. falciparum *to aid the process of drug target identification and distinction between targets suitable for prophylaxis, therapy or both. For this purpose, available resources were browsed to construct a multi-compartment metabolic network as complete as currently possible. The resulting set of biochemical reactions and transport processes was adapted to obtain a consistent network, which was validated by flux balance simulations to fulfill metabolic processes, such as hemoglobin digestion, that are known from the literature to occur in *P. falciparum*. Subsequently, a modified version of the method proposed by Shlomi *et al. *[25] was applied to calculate metabolic flux distributions specific for the individual life cycle stages with respect to the expression status of corresponding genes. In addition, *in silico *knock-out studies were performed with flux balance analysis to identify metabolic reactions that are essential for parasite development. This approach outperformed the previously proposed choke-point analysis [23,24] in terms of specificity, accuracy and precision when evaluated with a set of known targets. Predicted target reactions were further examined with regard to homology to human enzymes, functional similarity to therapeutic targets in other organisms and their predicted suitability for prophylaxis and disease treatment.

## Results

### Compilation of metabolic networks for P. falciparum and human erythrocytes

A metabolic network for *P. falciparum *has been compiled in a semi-automated manner based on data from the online databases Malaria Parasite Metabolic Pathways (MPMP) [26], BioCyc [27], KEGG [28], Reactome [29], BRENDA [30], and the Transport Classification Database [31]. Subsequently, the compiled metabolic network was tested for consistency. This procedure ensured among others that there are no reactions with generic stoichiometry and no reaction duplets. In order to link related reactions, corresponding metabolites were ensured to have the same level of specificity. For example, highly specific metabolites such as *α*-D-Glucose were replaced by a more general metabolite (D-Glucose) if both forms existed within the network. The functionality of this network was tested by using flux balance analysis (FBA) to calculate flux distributions that produce metabolites thought to be important for parasite development, including phospholipids, amino acids, nucleotides and cofactors. In this context MinModes [32] were calculated for each of these metabolites, which are basically minimal steady state flux distributions that allow for the production of a certain metabolite. In case no MinMode could be calculated, i. e. no path exists that converts external nutrients into the metabolite of interest, precursors of the metabolite (as defined by metabolic maps of KEGG and MPMP) were consecutively checked with respect to their producibility until one was found that is producible, thus revealing missing reactions and transporters. Endproducts and intermediates of pathways known from the literature to occur in *P. falciparum*, e. g. glycolysis, pentose phosphate cycle, hemoglobin degradation and glutathione conjugate export, were examined in the same way. This process revealed that mostly intracellular transporters were missing in the network that results from the data of the different sources. Reactions that had to be added include tRNA ligases in the mitochondrion and apicoplast, DNA and protein synthesis reactions, and the biosynthesis of un-saturated fatty acids. The resulting metabolic network is available in SBML format (see Additional file 1 and Table 1 for more details).

**Table 1:** Overview of assembled metabolic network for P. falciparum and the human erythrocyte

Organism	Reactions	Transporters	Metabolites	Genes	Compartments
Plasmodium falciparum	998	377	1622	579	6
Human erythrocyte	349	88	566	-	1

As predictions are thought to improve when the parasite is integrated into its natural environment, we decided to additionally compile the metabolic network for the human erythrocyte, which is the best studied system among the host cells due to its reduced metabolic capacity and relatively simple availability. For the erythrocyte multiple metabolic networks have been published that comprise the cell's core metabolism, including glycolysis, pentose phosphate pathway, glutathione metabolism and adenine nucleotide metabolism (see [33,34] and contained references). However, these networks lack metabolic pathways providing cofactors such as thiamin diphosphate and pyridoxal phosphate that are required for full cellular function. Therefore, we compiled the metabolic network for human erythrocytes from available online resources to obtain a network that is as complete as possible. As a main source served the enzyme database BRENDA [30], from which all EC numbers were extracted that are assigned to enzymes of human erythrocytes. Furthermore, the human red blood cell database [35], which catalogues the proteome of human red blood cells, was queried for enzymes and their EC numbers to complement those obtained from BRENDA. Subsequently, the EC numbers were used to extract reactions from the KEGG database. Transport processes were added as suggested by the Malaria Parasite Metabolic Pathways website [26] as well as from literature [36-50]. The network was subsequently checked for its consistency and its ability to produce essential metabolites as described above (see Table 1 for further details and Additional file 2 for SBML file).

### Life cycle gene expression profiles

This work intends to deduce stage-specific metabolism for all stages of the life cycle of *P. falciparum *on the basis of gene expression profiles. Since especially the liver stage in the human host is experimentally difficult to assess, we additionally analyzed gene expression data, covering the full life cycle of *P. yoelii *a close relative of *P. falciparum *causing malaria in rodents. Metabolism predictions based on these expression data are assumed to give an insight into the metabolic behavior of *P. falciparum *for those stages lacking large scale expression profiling. Gene expression data has been gathered from five publications (see Additional file 3): (a) a genome-scale transcriptome analysis of *P. falciparum *covering every hour of the intraerythrocytic developmental cycle (IDC) by Bozdech *et al. *[5], (b) a genome-scale transcriptome analysis including nine time points of *P. falciparum *blood stage (early and late ring stage, early and late trophozoite, early and late schizont, merozoite, and gametocyte) and mosquito stage (salivary gland sporozoite) by Le Roch *et al. *[6], (c) a cDNA library obtained from liver stage expression analysis of the rodent malaria pathogen *P. yoelii *40 hours post infection by Sacci *et al. *[7], (d) a data set from Tarun *et al. *[9], including seven samples extracted from *P. yoelii*, covering three time points during the liver stage (24 h, 40 h, and 50 h post-infection), two time points during the mosquito stage (10 days (midgut) and 15 days (salivary gland) after blood meal), and two samples from blood stages (schizont and mixed blood stages), and (e) a genome-scale transcriptome analysis by Daily *et al. *of 43 samples derived from the blood of *P. falciparum *infected patients which contain mostly ring stages of the parasite [8].

In a first step, the accordance of these data sets was checked for corresponding stages. For this purpose the expression status was determined for each gene of every expression sample as described in the Methods section. Differences between the gene expression samples were then assessed with a simple and intuitive distance measure, the normalized Hamming distance, which gives for two strings of equal length the rate of positions at which the corresponding symbols are different:

$$dist(x,y)=\frac{\sum_{x_{i}\neq y_{i}}1}{\sum_{i}1}$$

In our case the symbols are binary values describing the expression state of a gene (**1: **gene is expressed; **0: **gene is not expressed) and thus the numerator is the sum of genes with different expression status and the denominator is the total number of common genes in two samples. The resulting Hamming distance matrix for all gene expression sample pairs is shown in Additional file 4.

The distance matrix reveals that samples of the same data set covering consecutive time points tend to be more similar compared to those covering non-consecutive time points, especially samples of the Bozdech or the Le Roch data set. Furthermore, similar gene expression patterns can be observed for samples of different data sets that correspond to the same life cycle stage, e. g. Le Roch ring stage samples and Bozdech samples extracted during hour 1 to 20 after erythrocyte infection. In addition, the distance matrix agrees well with the observation by Daily *et al. *that the *in vivo *ring stage expression samples can be ascribed to three clusters [8]: starvation response accompanied by metabolism of alternative carbon sources (cluster 1), active growth based on glycolytic metabolism (cluster 2) and environmental stress response (cluster 3). The distance matrix confirms that samples of the same clusters are more similar in terms of expressed enzymes. Only a fraction of cluster 3 samples exhibits similarity to cluster 1 samples. Moreover, it shows that samples of cluster 2 are similar to those samples of the Bozdech and Le Roch data sets which correspond to early or very late blood stages. In general, Hamming distances are larger when samples belong to different data sets. This can be ascribed to one or more of the following issues (i) different methods have been used to analyze gene expression (microarray analysis vs. cDNA library), (ii) experiments are conducted with different organisms (*P. falciparum *vs. *P. yoelii*) and (iii) for the individual data sets different criteria have been applied to classify genes as absent or present.

### Gene expression data mapped onto metabolic pathways

Mapping gene expression data onto metabolic networks may uncover active pathways for each stage and metabolic differences between the individual life cycle stages. In this context, we calculated the ratio of expressed genes per pathway (as defined by KEGG) for each gene expression sample. This ratio was then used to group pathways with respect to co-expression of involved enzymes to detect whether there are patterns in the activity of pathways as a function of the individual developmental stages. Since the blood stage is the only phase covered by all data sets, samples extracted during the IDC were used to cluster the pathways (see Additional files 5, 6 and 7).

Three pathway clusters can be identified for the Bozdech and the Le Roch data sets, which roughly correspond to each other (see green, blue, and yellow bar in Additional file 5 and 6). The green clusters, which have 10 pathways in common (e.g. glycolysis, glycerophospholipid metabolism and porphyrin metabolism), contain pathways where only a fraction of pathway associated genes are expressed during the IDC with a maximum of expressed genes during late trophozoite and early schizont stage. Pathways associated with the blue clusters (common pathways: fatty acid biosynthesis, lipoic acid metabolism, vitamin B6 metabolism and one carbon pool by folate metabolism) show gene expression almost exclusively during late trophozoite and early schizont stages of the IDC, while those of the yellow clusters (common pathways: pentose phosphate pathway, inositol phosphate metabolism) exhibit many expressed genes during all phases of the intraerythrocytic developmental cycle. Notably, during the gametocyte stage (Le Roch data set) a high number of genes are expressed in all pathways and in contrast only few genes during the invasive stages (Le Roch data: sporozoite, merozoite). Pathway clusters as described above for the blood stage are not as apparent for the Tarun data, since this stage has not been analyzed as detailed. Nevertheless, certain pathways group together as seen for the Bozdech and Le Roch data sets (see colored bars in Additional file 7). Daily and coworkers found that their ring stage expression samples can be divided into three clusters: starvation response accompanied by metabolism of alternative carbon sources (cluster 1), active growth based on glycolytic metabolism (cluster 2) and environmental stress response (cluster 3). These clusters can be found as well when expression samples are mapped to metabolic pathways as shown in Additional file 8. In samples of cluster 1 almost all enzymes are expressed in each of the considered pathways, suggesting that required metabolites cannot be obtained in sufficient amounts from the host and thus the parasite has to activate all possible pathways in order to obtain these metabolites. In contrast, less enzymes are expressed in samples corresponding to cluster 2. Here, enzymes of sugar metabolism related pathways like glycolysis, pentose phosphate pathway and glycerophospholipid metabolism (blue bar) are mostly activated. Similar expression patterns can be observed for cluster 3 samples. However, more enzymes are expressed in certain pathways such as fatty acid synthesis, thiamine metabolism and ubiquinone biosynthesis (pink bar).

In general, the majority of metabolic pathway genes is expressed during starvation (Daily data) or late trophozoite and early schizont stage where reproduction of biomass is at its peak [43]. Pathways showing similar patterns of activation and suppression are presumably co-regulated. Especially in the case of purine and pyrimidine biosynthesis this is plausible, since both nucleotide types are needed in large amounts at the same time during DNA replication and mRNA synthesis. Likewise the expression of genes associated with fatty acid biosynthesis, pyruvate metabolism, and lipoic acid metabolism seems to be coupled, since only few genes are expressed during blood stages but significantly more during liver stages. Most of the reactions assigned to these three KEGG pathways are assumed to take place in the apicoplast and are linked to each other. When fatty acid demand is fully satisfied by import, which is suggested for the blood stage by findings of Vaughan *et al. *[51], neither the precursor acetyl-CoA (pyruvate pathway) nor lipoic acid, a co-factor of pyruvate dehydrogenase catalyzing acetyl-CoA synthesis, are needed within the apicoplast. Thus, it seems reasonable that these three pathways, which cluster together when considering the Bozdech dataset, are co-regulated.

### Metabolic flux predictions for different life cycle stages

**Figure 1:**
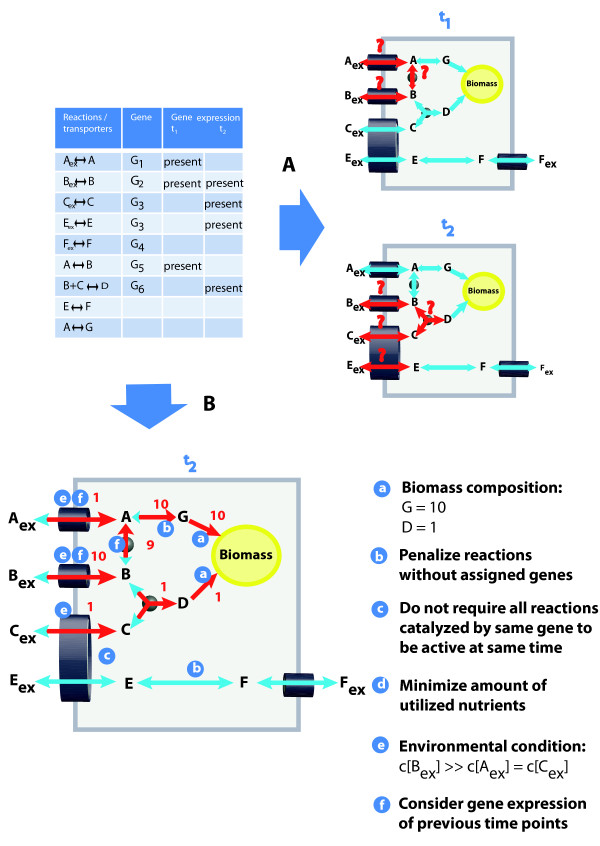
**Illustration of presented flux balance approach to predict life cycle specific metabolism**. Given the gene expression data (blue table) flux distributions (red arrows) within the shown example metabolic network (blue arrows) can be inferred for time points *t*_1 _and *t*_2 _as depicted in (A). However, neither flux direction nor flux strength can be deduced from gene expression alone (indicated by question marks next to flux arrows). The set of all possible flux distributions that are consistent with the gene expression data can be reduced by knowledge about target fluxes such as biomass production (i). Reactions that are not supported by genome annotation might represent errors in the network assembly. Therefore it is desirable to prevent the usage of such reactions in calculated flux distributions (ii). An enzyme or a transporter that is able to process different metabolites does not necessarily convert all substrates at same rates. If one reaction product is not converted further by subsequent enzymes, it accumulates and as a consequence the net production rate is close to zero, even if the gene is expressed and substrate is available (iii). The flux solution space can be narrowed down further when assuming that biomass production is achieved with a minimal amount of nutrients (iv), which are of varying availability (v). Gene products can be present within a cell, even when the gene transcript is not detectable, as proteins appear later than the corresponding mRNA and protein degradation might be delayed compared to mRNA degradation. Considering proteins to be present whose transcript was detectable during a previous time point (vi) presumably reflects the actual cellular status better than taking only the current transcription snapshot into account. The flux distribution calculated by our flux balance approach, which incorporates all these issues, is shown in (B).

Gene expression data give an impression of the metabolic status of a cell, as has been shown in the previous section. However, gene expression data alone are not sufficient to deduce metabolic fluxes. Simply assuming a correlation between gene expression levels and metabolic fluxes is not appropriate, since it has been shown that neither mRNA levels and protein levels always correlate well [52,53] nor enzyme levels and corresponding flux rates of catalyzed reactions [54,55]. Furthermore, expression data is not always available for all genes of interest, or in some cases, there are no genes assigned to metabolic reactions at all, if they are rather inferred from metabolic context than based on direct biochemical evidence. In addition, from gene expression data alone no information can be gained with regard to which nutrients are imported into the cell and which metabolites are secreted. Flux balance approaches provide a means to predict flux distributions despite such obstacles. Flux values can be predicted even for those reactions where no gene expression data is available, as fluxes are not independent due to the assumed steady state condition, which requires the sum of fluxes producing a metabolite to equal the sum of fluxes consuming it. A reasonable objective function describing the cellular goals is essential to obtain meaningful flux distributions with FBA. In the case of *P. falciparum *the overall goal is reproduction, and thus biomass production on the metabolic level. At the same time the parasite has an interest to keep its host cell alive for the period of replication which sets an upper limit on the replication rate. Depending on how well the organism of interest is already studied, the objective function might neglect certain metabolically important issues, e. g., a metabolite essential for reproduction that has not been noticed yet. On this account, we decided to apply an approach that combines FBA and gene expression information to calculate flux distributions for the different parasitic life cycle stages. The integration of expression data ensures that metabolic aspects not captured by our objective function are nevertheless represented in the calculated flux distributions. Our approach is based on a FBA approach proposed by Shlomi *et al. *[25], which maximizes the number of reactions whose activity is consistent with the expression state of the corresponding genes. We made several modifications to this technique (see Figure 1): (a) Constraints were added to ensure the production of important biomass precursors such as phospholipids, which are needed for parasite reproduction (see Additional file 9). These target metabolites might vary depending on the stage covered by the given gene expression sample. DNA synthesis is important for stages aiming at reproduction, like trophozoites. In contrast, for invasive forms of the parasite (sporozoite, merozoite, etc.) this is not an issue and therefore not considered in flux calculations corresponding to these stages. Furthermore, blood stages need to incorporate heme from hemoglobin degradation into hemozoin molecules in order to prevent intoxication and cell lysis. This makes hemozoin a target metabolite for these stages, but not for liver or mosquito stages where hemoglobin is not present. Targeted deletions of critical fatty acid synthesis enzymes in *P. yoelii *and *P. falciparum *suggest that fatty acid synthesis in the apicoplast is only essential during late liver stage [51]. This implies that pyruvate dehydrogenase (PDH), which provides fatty acid synthase with acetyl-CoA, is not essential during any other stage and thus lipoate, a cofactor of PDH, is as well dispensable in the apicoplast. Since PDH is the only enzyme in the apicoplast that requires lipoate, we demanded lipoate biosynthesis in this organelle only during late liver stage. (b) Constraints were added to minimize the number of active reactions whose existence is not supported by genome annotations. This is useful as especially data from the BioCyc database contain reactions without any associated gene that are nevertheless declared to be present in *P. falciparum *to fill gaps in pathways. (c) The original approach proposed by Shlomi *et al. *seeks all reactions catalyzed by the same enzyme to be simultaneously active if the corresponding enzyme is expressed. As this might not always be appropriate, our method only tries to assign a non-zero flux to at least one of these reactions. (d) The network is forced to fulfill its tasks with a minimal amount of externally supplied metabolites to account for the limited amount of nutrients available in the host cell that has to be shared among the offspring. More details about the approach are provided in the Methods section.

The approach was applied to each of the gene expression samples extracted from different time points of parasite development (see Additional file 3) to predict respective metabolic fluxes during these time points. In order to compare the resulting flux distributions for the individual time points, normalized Hamming distances were computed for all flux distribution pairs (see Additional file 10). In general, the calculated flux distributions are more similar to each other (max. Hamming distance: 0.188) than the corresponding gene expression samples (max. Hamming distance: 0.718). However, similarity patterns as observed for the gene expression data are also detectable for the flux profiles. For example, flux profiles corresponding to consecutive time points during the blood stage are more similar than those of non-consecutive time points (see Bozdech data; trophozoite stage vs. schizont) and flux profiles corresponding to *in vivo *expression profiles of the same cluster tend to be more similar than those of different clusters.

As experimental data on intra-parasitic fluxes are not available, we checked the plausibility of calculated flux distributions by comparing predicted exchange fluxes between host and parasite with available experimental observations.

### Metabolite exchange with host

During its life cycle, *P. falciparum *is exposed to different environments in its hosts (human liver, human erythrocyte, mosquito gut, mosquito salivary gland) and thus has to deal with different nutrient supply. It has been shown for *Trypanosoma brucei *[56] that this parasite is able to remodel its metabolism depending on available carbon sources. It is very likely that *P. falciparum *utilizes likewise varying parts of its enzyme equipment as a function of available nutrients, which results in different sets of metabolites transported through the parasite's plasma membrane during the individual developmental stages.

Additional file 11 gives an overview of imported (red) and excreted (green) metabolites during the individual life cycle stages, as predicted by our FBA method when considering only the metabolic network of the parasite, without any further information about the parasite's environment. Glucose, the main energy source for *P. falciparum *during the IDC, is converted via glycolytic enzymes to lactate, which is then excreted [57]. This phenomenon is recovered by our simulations for most of the samples extracted during the blood stage. For multiple gene expression samples amino acids are predicted to be exported, which contrasts the naive assumption that all available amino acids are needed for protein biosynthesis. In fact, the parasite digests up to three quarters of the host hemoglobin during the IDC to provide amino acids for protein synthesis, to gain space in the host cell for further growth [58] and to maintain the osmotic stability of the host cell [59]. Excess amino acids not incorporated into the parasite's proteome are exported out of the infected host cell [60] and is therefore in accordance with the predictions. Experiments have shown that *P. falciparum *relies on exogenous supply of isoleucine, the only amino acid that is not present in hemoglobin [61]. For all blood stage related samples isoleucine uptake has been correctly predicted. Moreover, in concordance with experiments by Kirk and Saliba [62], coenzyme A precursor pantothenate is imported in all our simulations. According to our predictions the parasite satisfies its demand for NAD+ precursors mostly with the import of nicotinamide, but also with nicotinate import, which is consistent with experimental findings [43] that suggest *P. falciparum *to be dependent on exogenous supply of these metabolites. The parasite possesses an antioxidant defense system, which involves glutathione to eliminate peroxides and toxic substances resulting in the formation of glutathione disulfate and glutathione conjugates that are subsequently either regenerated or removed from the cell [63]. All predicted flux distribution correctly suggest the export of glutathione conjugates.

However, there are predicted transport processes that are not in agreement with literature knowledge. For example, phosphate is mostly predicted to be exported, while it is reported to be taken up [64]. Likewise, ATP is imported rather than exported to support the host as proposed by Kanaani and Gins-burg [65] and hemoglobin is predicted to be taken up during liver stages where no hemoglobin is present. Furthermore, purine precursors are imported mostly in the form of inosine, while hypoxanthine is exported, despite being available in higher concentrations in the blood plasma than the other purine precursors. These discrepancies indicate that the applied model, which assumes that all nutrients are available without limits, is not appropriate and thus further constraints reflecting the parasites metabolic environment are needed in order to obtain more reliable flux predictions.

### Improved metabolic flux predictions for parasitic blood stage

A model which does not define the parasite's environment might results in certain incorrect predictions as has been shown in the previous section. Therefore, we tried to improve predictions by integrating additional information about the parasite's host and available nutrients. These improvements could only be implemented for the blood stage of the parasite to which most of the current knowledge about the malaria pathogen correspond due to its simple experimental accessibility and the fact that among the host cells the erythrocyte is the best studied system. We combined the parasite's metabolic network with that of the erythrocyte and forced the combined network to produce in addition metabolites important for erythrocyte metabolism. Furthermore, constraints reflecting knowledge about the blood stage were incorporated into the calculations. For example, the parasite was forced to consume at least a certain amount of hemoglobin and glucose, since both metabolites are known to be consumed by the parasite in large amounts [62,66]. Up-take limits were set for several metabolites, including purine precursors, sugars, cofactors and phospholipid headgroup precursors, which are proportional to the respective blood plasma concentrations as listed in the Human Metabolome Database http://www.hmdb.ca/. In addition, constraints were added that ensure trapping of purine precursors as well as choline and ethanolamine within the parasite due to phosphorylation [67,68]. Observations by Mehta *et al. *[69] indicate that *P. falciparum *is able to repress glycolysis of erythrocytes by inhibiting their glycolytic enzymes phosphofructokinase and pyruvate kinase. In concordance with these observations both host enzymes were inhibited in flux calculations that correspond to stages subsequent to the ring stage of the IDC.

For a certain fraction of *P. falciparum *genes, the transcriptome was found to correlate better with the proteome of the following than the current stage [70]. For this reason, considering only the gene expression snapshot of a single time point does not give a complete picture on the actual present proteome. Including the gene expression status of previous time points into the calculations should therefore further improve flux predictions. We modified our algorithm in such a way that genes were considered to be expressed, if a transcript is either present during the time point of interest or during a time span of approximately 12 hours prior to this time point (concerns 12 preceding samples of Bozdech data set and 2 preceding samples of Le Roch data set). Since the time intervals between samples covering the liver or the mosquito stage are larger, this modification was only applied to flux predictions for the blood stage.

**Figure 2:**
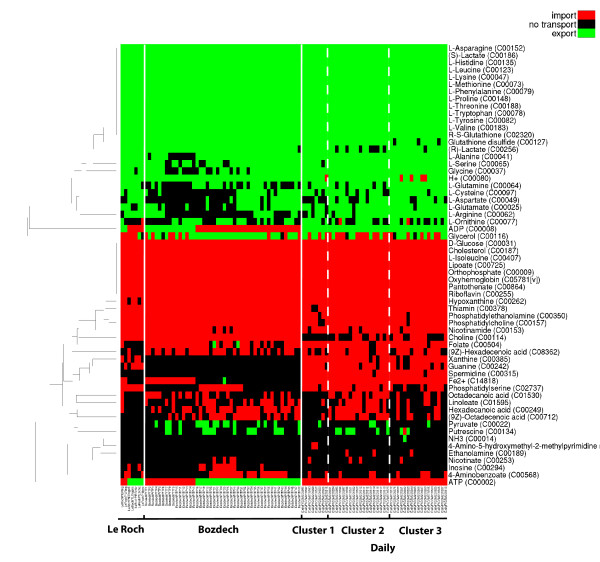
**Predicted host parasite metabolite exchanges using improved algorithm**. Flux distributions have been predicted with our improved flux balance approach (see Figure 1) for each time point of the intraerythrocytic developmental cycle for which a gene expression profile exists. Simulations were conducted on the basis of the combined metabolic network of parasite and host and additional constraints reflecting knowledge about the blood stage. Furthermore, the expression status of genes during preceding time points was considered for the flux calculations. Resulting metabolite exchanges between host and parasite are depicted in this figure. Red matrix entries represent metabolites that are predicted to be imported into the parasite, while green matrix entries represent metabolites secreted into the host compartment.

Metabolites predicted to be exchanged between host and parasite when using the improved algorithm (combination of host and parasite network, additional constraints reflecting knowledge about the blood stage as well as consideration of gene expression status of preceding time points; see Additional file 12) are in considerably better accordance with experimental findings (see Figure 2 and Table 2) than before. In contrast to the previous predictions derived from the unmodified algorithm, purine precursors and choline are only imported and not secreted, while proteinogenic amino acids (except for isoleucine) are only exported. In our flux calculations hemoglobin is assumed to be digested at higher rates during trophozoite and schizont stages of the IDC, which releases iron that can be utilized by the parasite [71-73]. When the host's glycolytic enzymes phosphofructokinase and pyruvate kinase are inhibited (trophozoite and schizont stages), ATP can no longer be obtained from the host. Thus, the parasite needs to produce more ATP on its own, which requires the import of additional phosphate. Moreover, in order to ensure host survival for a certain time period the parasite provides the host with ATP, which is in accordance with findings by Kanaani and Ginsburg [65].

**Table 2:** Metabolite exchanges between parasite and host: prediction vs. experiment

Metabolite	Predicted	Experiment	Comment	References
Glucose	Uptake	uptake		[57,128,129]
Lactate	Secretion	secretion		[57,128,129]
Hemoglobin	uptake	uptake		[57,128]
Amino acids	Secretion	secretion		[57,128,129]
Isoleucine	uptake	uptake		[57,128,129]
Nucleosides/nucleobases	uptake	uptake		[128-130]
Choline	uptake	uptake		[68,128,129,131]
Ethanolamine	almost no exchange	uptake	No gene assigned to ethanolamine transporter	[128,132]
Phosphatidylethanolamine	uptake	uptake		[128,132]
Phosphatidylserine	uptake	uptake		[128]
Phosphate	uptake	uptake		[64,128,129]
Pantothenate	uptake	uptake		[62,128,129]
Nicotinamide/nicotinate	uptake	uptake		[43,128]
Folate	uptake	uptake		[128,129]
4-aminobenzoic acid	uptake	uptake		[128,129]
ADP	uptake/secretion	uptake	secretion in early blood stage when ATP can still be salvaged from host	[65,128,129]
ATP	uptake/secretion	secretion	uptake in early blood stage when host glycolysis is not repressed yet	[65,128,129]
Glycerol	uptake/secretion	uptake/secretion		[128,129]
Glutathione conjugate	secretion	secretion		[63,128,129]
Ornithine	mostly secretion	secretion		[43]
Cholesterol	uptake	uptake		[128]
Formate	no exchange	secretion	Erythrocyte lacks reactions and transport processes to dispose of metabolite	[128,129]
Fe2+	no exchange/uptake	uptake		[128,129]
H+	Mostly secretion	secretion		[128,129]
HC03-	no exchange	secretion	Erythrocyte lacks reactions and transport processes to dispose of metabolite	[128,129]

In general, flux profiles derived from *in vitro *(Bozdech and Le Roch data set) and *in vivo *gene expression samples (Daily data set) are fairly similar in terms of exchanged metabolites between host and parasite, especially those corresponding to the active growth like cluster (cluster 2). One main difference is that more purine precursor types are predicted to be imported for the *in vivo *samples. The main differences between *in vivo *clusters is the absence of predicted fatty acid and choline import for samples of the starvation like cluster (cluster 1). This could be attributed to environmental conditions that result in a lack of nutrients required for parasite growth. Cell membrane production is then either not possible or not the main focus and thus the import of membrane precursors like fatty acids and choline is not required.

There are still some metabolites whose predicted transfer direction through the parasite's plasma membrane does not meet literature knowledge. For example, it has been found that arginine contained in the medium is imported and depleted by the parasite, resulting in ornithine which is then excreted [43]. In contrast, our simulations suggest arginine export. Since these observations were made at excessively high arginine concentrations, further experiments are needed to examine arginine transport under physiological conditions. Our predictions are more in line with experiments by Liu et al. [61] which indicate that hemoglobin degradation satisfies the parasite's demand for all amino acids during the blood stage except for isoleucine and thus isoleucine is the only amino acid required to be taken up. Other predicted metabolite transports that contradict literature knowledge are those of formate, hydrogen-carbonate and ethanolamine. Almost no transport is predicted for ethanolamine, because the transport process is not associated with any gene and therefore our algorithm avoids to assign non-zero fluxes. However, this is not the case for formate and hydrogen-carbonate, but rather missing reactions and transport processes in the erythrocyte that are needed to dispose of these metabolites. Thus, predictions can still be further improved when more knowledge is available on the particular developmental stages as well as the host cell.

Counting for each metabolic reaction and transport process in how many stage-specific flux distributions it is predicted to be active when using the improved algorithm (see Additional file 13), reveals that nearly 350 reactions carry a non-zero flux in all 96 flux profiles that are derived from gene expression samples extracted during the IDC. In comparison to these globally active reactions, others that are present during fewer time points can be considered as more stage-specific.

### Calculated fluxes mapped onto metabolic pathways

In order to explore the predicted flux distributions derived from the improved algorithm on the level of metabolic pathways, we again performed a mapping. This time, instead of counting expressed genes per KEGG pathway, we counted reactions carrying non-zero fluxes, to assess whether a pathway is active or not and wether there are changes during the IDC (see Additional files 14, 15 and 16). Due to the integration of gene expression states of preceding time points into the calculations more enzymes are considered to be present than are suggested by the gene expression sample of the time point of interest. In general, for consecutive time points of gene expression sample extraction the number of active reactions per pathway does not vary as much as the number of expressed genes per pathway (compare to Additional files 5 and 6). Therefore, fewer metabolic changes are predicted over the course of the IDC than are suggested by the gene expression data. Nevertheless, time dependent activity variation can be observed for certain pathways. For example, fewer reactions corresponding to folate biosynthesis are predicted to be active during the ring stage than during subsequent stages. In contrast, for the citric acid cycle as well as sphingolipid metabolism an increase in active reactions is predicted during schizont and early ring stage.

Predictions derived from *in vivo *gene expression samples show mainly similar pathway activity levels. Samples of the starvation-like cluster (cluster 1) vary from the others only with respect to lipid metabolism related pathways, which have a higher fraction of active reactions (especially the fatty acid biosynthesis pathway), and energy metabolism. Less reactions of the glycolytic pathway are active while more reactions of the citric acid cycle are active compared to cluster 2. The same holds true for some samples of the stress response-like cluster (cluster 3).

However, for some pathways the flux distributions vary substantially from the reported expression profiles. For instance, according to our flux predictions synthesis of terpenoids (part of steroid synthesis pathway) takes place during the complete IDC, while gene expression data suggest this pathway to be active only during the second half of the IDC. This is due to the chosen set of target metabolites required to be produced during the individual stages (see Additional file 9), which includes isopentenyl diphosphate. This might not be appropriate for all time points.

### Determination of fluxes with higher reliability

For each stage of the parasite's developmental cycle, we determined so-called consensus reactions that are predicted to be active for all gene expression samples covering the same stage. These reactions are more likely to actually occur during a certain stage, since they are derived from different data samples. In order to identify consensus reactions we grouped all calculated flux profiles into 13 sets (LS24, LS40, LS50, eRing, lRing, eTropho, lTropho, eSchiz, lSchiz, Mero, Gameto, ooSporo, sgSporo; see Additional file 3) with respect to represented stages and determined those reactions that carry a non-zero flux in all flux profiles of the same set. For all stages of the intraerythrocytic developmental cycle those flux profiles were considered that were calculated with the improved algorithm.

For each blood stage and each metabolic pathway the fraction of consensus reactions per total number of pathway reactions was computed to uncover the distribution of consensus reactions among pathways (see Additional file 17). To get an impression of how many consensus reactions are shared between the different stages, this fraction was also computed for those consensus reactions that two sets have in common. In several pathways the same reactions are active during the whole IDC (e. g. ubiquinone biosynthesis and terpenoid biosynthesis). These are mostly biosynthesis pathways where alternative routes are rare and the endproducts are considered to be essential. Therefore, the network is forced to produce them, which results in a large overlap between the predicted fluxes through these pathways for all time points. By contrast there are pathways where more accordance can be observed for trophozoite and schizont stages (e.g. glutathione metabolism, pentose phosphate pathway). This is mainly due to the fact that consensus reactions for the early and late ring stage are derived from many more samples than those for other stages. Especially the heterogeneous *in vivo *expression samples contribute to a low number of consensus reactions for these stages. The citric acid cycle is one of the few pathways where a certain level of agreement exists between ring and schizont stages.

### Search for essential reactions as potential drug targets

Beside the prediction of stage-specific metabolic fluxes the goal of this work is the identification of novel drug targets in *Plasmodium falciparum*. For this purpose flux balance simulations were conducted to analyze the compiled metabolic network with regard to essential reactions required for the production of important cellular metabolites. Enzymes catalyzing such reactions represent potential drug targets, since their inhibition would result in a shortage of required metabolites and thereby in impaired development. Knock-outs were simulated with a simple FBA approach neglecting gene expression information. No assumptions were made about the cellular environment and nutrient uptake was not restricted, since such information is not available for all stages. Reactions were successively constrained to carry no flux, while the network was forced to produce all metabolites that are assumed to be essential during any developmental stage (all metabolites listed in Additional file 9). If no solution could be found to this problem the reaction was assumed to be essential. For more details see the Methods section. As not all metabolites are equally important during all time points of parasite development, e. g. DNA building blocks are not required to be synthesized during the merozoite stage, a reaction detected by this means might not be globally essential, but rather during those time points when the particular metabolite requiring the activity of the reaction is indispensable. On the other hand, certain reactions that are not detected as essential might be incorrectly classified, if nutrients are actually not available in sufficient amounts during all time points, which conflicts the simulation assumption. In this case essential metabolites cannot be derived from imported precursors, but rather need to be completely synthesized, making additional reactions indispensable. Thus, all reactions identified as essential by this approach are essential during at least one developmental stage, but not all reactions that are essential during any stage are necessarily recovered. To evaluate our results we used as our gold standard a set of 57 enzymes that were shown to have antimalarial effects upon inhibition or silencing (see Table 3).

**Table 3:** Gold standard set of essential enzymes

EC	Reaction	Choke-points	Found by FBA	Comment	Reference
1.1.1.205	IMP dehydrogenase	Yes	No	Precursor import (adenosine, hypoxanthine, inosine)	[133]
1.1.1.267	1-deoxy-D-xylulose-5-phosphate reducetoisomerase	Yes	Yes		[84]
1.3.1.9	enoyl-ACP-reductase	Yes	Yes		[134-136]
1.3.3.1	Dihydroorotate oxidase	No	Yes		[92-95,137]
1.3.99.1	Succinate dehydrogenase	No	No	Presumably unspecific off-target effects	[75]
1.5.1.3	Dihydrofolate reductase	No	No	Necessary to block all reactions catalyzed by the enzyme	[138-140]
1.6.5.3	NADH dehydrogenase (ubiquinone)	No	No	Presumably unspecific off-target effects	[74]
1.8.1.7	glutathione reductase	Yes	Yes		[141,142]
1.8.1.9	thioredoxin reductase	Yes	Yes		[143,144]
1.10.2.2	cytochrome c reductase	Yes	Yes		[145]
1.15.1.1	superoxide dismutase	Yes	Yes		[146]
1.17.4.1	Ribonucleoside-diphosphate reductase	No	Yes		[147-149]
2.1.1.100	Protein-S-isoprenylcysteine-O-methyltransferase	Yes	Yes		[150,151]
2.1.1.103	Phosphoethanolamine methyltrans-ferase	Yes	No	Presumably unspecific off-target effects	[77,79]
2.1.1.45	Thymidylate synthase	No	Yes		[152,153]
2.1.1.64	3-Demethylubiquinone-9,3-O-methyltransferase	Yes	Yes		[154]
2.3.1.24	sphingosine-N-acyltransferase	Yes	Yes		[155]
2.3.1.37	delta-aminolevulinate synthase	Yes	Yes		[99,101]
2.3.1.41	3-Oxoacyl-[acyl-carrier protein] synthase	Yes	Yes		[156,157]
2.3.1.50	serine-palmitoyl transferase	No	Yes		[155]
2.4.2.1	Purine-nucleoside phosphorylase	Yes	No	Precursor import (hypoxanthine, xanthine)	[158]
2.4.2.8	Hypoxanthine phosphoribosyltransferase	Yes	No	Necessary to block all reactions catalyzed by the enzyme	[159-161]
2.5.1.15	Dihydropteroate synthase	Yes	No	Precursor import (folate)	[162-164]
2.5.1.16	Spermidine synthase	Yes	No	Precursor import (spermidine)	[165]
2.5.1.18	Glutathione transferase	Yes	Yes		[166-169]
2.5.1.19	3-Phosphoshikimate 1- carboxyvinyltransferase	Yes	No	Precursor import (4-aminobenzoate, folate)	[170]
2.5.1.58	Farnesyl-diphosphate Farnesyltransferase	No	Yes		[171]
2.7.1.32	Choline kinase	No	No	Presumably unspecific off-target effects	[78]
2.7.8.3	Ceramide-cholinephosphotransferase	Yes	Yes		[155]
3.3.1.1	S-adenosyl-l-homocysteine hydrolase	Yes	Yes		[172-175]
3.4.11.1	leucine aminopeptidase	Yes	No	Precursor import (amino acids)	[176]
3.4.14.1	dipeptidyl aminopeptidase 1	No	No	Precursor import (amino acids)	[177]
3.4.23.38	plasmepsins (aspartic acid proteases)	No	No	Precursor import (amino acids)	[178,179]
3.4.23.39	plasmepsins (aspartic acid proteases)	No	No	Precursor import (amino acids)	[178,179]
3.5.1.89	N-acetyl glucosaminylphosphatidyli-nositol deacetylase	No	Yes		[180]
3.5.2.3	dihydroorotase	Yes	Yes		[96,137]
3.5.4.4	Adenosine deaminase	Yes	No	Precursor import (hypoxanthine, inosine, xanthine)	[181,182]
4.1.1.17	Ornithine decarboxylase	No	No	Precursor import (spermidine, putrescine); alternative reaction R01152	[183-185]
4.1.1.23	Orotidine-5'-phosphate decarboxylase	Yes	Yes		[89-91,97,98]
4.1.1.50	Adenosylmethionine decarboxylase	No	No	Precursor import (spermidine)	[186]
4.1.2.13	Fructose-bisphosphate aldolase	Yes	No	Presumable main antimalarial effect is impaired host cell invasion	[187,188]
4.2.1.1	carbonic anhydrase	Yes	Yes		[87]
4.2.1.24	Delta-aminolevulinic acid dehydratase	No	Yes		[100]
4.2.1.58-61	3-hydroxyacyl-ACP dehydratase	Yes	Yes		[189]
4.2.3.5	Chorismate synthase	Yes	No	Precursor import (4-aminobenzoate, folate)	[92]
4.4.1.5	Lactoylglutathione lyase	Yes	Yes		[190]
4.6.1.12	2-C-methyl-D-erythritol-2,4- Cyclodiphosphate-synthase	No	Yes		[86]
5.99.1.2	topoisomerase I	Yes	Yes		[191]
5.99.1.3	topoisomerase II	Yes	Yes		[192-194]
6.1.1.3	Threonine-tRNA ligase	Yes	Yes		[195]
6.1.1.7	Alanine-tRNA ligase	Yes	Yes		[196]
6.3.2.2	Gamma-glutamylcysteine synthetase	Yes	Yes		[144,197,198]
6.3.4.4	Adenylosuccinate synthetase	Yes	Yes		[199]
6.3.5.2	GMP synthetase	No	No	Necessary to block all reactions catalyzed by the enzyme; precursor import(guanine/guanosine)	[200]
6.3.5.5	Carbamoyl-phosphate synthase (glutamine-hydrolysing)	No	Yes		[88]
6.3.5.8	amino-deoxychorismate synthase	No	No	Precursor import (4-aminobenzoate, folate)	[128]
6.4.1.2	Acetyl-CoA carboxylase	Yes	Yes		[156]

The simulations identified 307 reactions that are essential for the parasite. This set includes reactions that correspond to 35 enzymes of our gold standard (see Table 3). Blocking all reactions catalyzed by the same gene product recovers two more targets (dihydrofolate reductase and hypoxanthine phosphorribosyltransferase) contained in our gold standard. Among the 20 remaining enzymes 13 are detected as essential by our method, if additionally transporters are constrained that import biomass precursors bypassing the respective enzyme. For example, inhibition of the spermidine transporter makes the spermidine synthase indispensable. These enzymes are therefore either not metabolically essential during all environmental conditions but only during those stages where particular nutrients are not present in sufficient amounts, or the respective parasitic transporters are not efficient enough. Furthermore, GMP synthase is detected if the import of guanine and guanosine is constrained and all reactions catalyzed by the enzyme are blocked. Ornithine decarboxylase is not found by our method, as a an alternative path exists to synthesize putrescine involving carbamoylputrescine amidase. Blocking this enzyme in addition to the spermidine and putrescine transporters makes ornithine decarboxylase indispensable. Carbamoylputrescine amidase is very likely not present in *P. falciparum*, since it was added in the BioCyc database to fill gaps in the putrescine biosynthesis pathway of which only one enzyme is associated with a gene. Succinate dehydrogenase and NADH dehydrogenase, which are not even detected when nutrient import is limited, are very likely false positives. For both enzymes of the mitochondrial electron transport chain antimalarial activity was demonstrated through inhibition with substrate analogs of ubiquinone [74,75]. As ubiquinone, the substrate of both enzymes, can be provided with electrons by several enzymes (succinate dehydrogenase, NADH dehydrogenase, dihydroorotate dehydrogenase, FAD-dependent glycerol-3-phosphate dehydrogenase, malate-quinone oxidoreductase), it is not quite obvious why inhibition of only one enzyme should result in impaired parasite development. One explanation could be that the ubiquinone analogs do not only inhibit one enzyme, but also others that use ubiquinone as a substrate. This is supported by the findings of Painter *et al. *[76], who discovered that an active mitochondrial electron transport chain might only be required for the disposal of electrons derived from dihydroorotate dehydrogenase, an essential enzyme for pyrimidine biosynthesis. They showed that parasites that express the corresponding yeast enzyme, which does not require ubiquinone as an electron acceptor, are insensitive to atovaquone, an inhibitor of the electron transport chain Complex III. This suggests that the antimalarial effect of ubiquinone analogs is rather due to either additional inhibition of the downstream Complex III or simultaneous inhibition of all enzymes providing ubiquinone with electrons (including dihydroorotate dehydrogenase), instead of the sole inhibition of succinate dehydrogenase or NADH dehydrogenase. Our simulations indicate further-more that neither sole inhibition of choline kinase nor sole inhibition of phosphoethanolamine methyltransferase kills the parasite, because phosphatidylcholine can be derived from choline as well as from ethanolamine [77]. The question remains whether these pathways are indeed able to fully compensate the loss of the alternative pathway, as both have been suggested to affect parasite growth [78,79]. However, the findings by Choubey *et al. *[78] can also be explained by unspecific inhibitor (analog of miltefosin) binding, resulting in the inhibition of both choline kinase and phosphoethanolamine methyltransferase [77]. Finally, the gold standard enzyme fructose-bisphosphate aldolase was not detected by our knock-out simulations. Our simulations do not confirm that aldolase is metabolically essential, since ATP can be generated from glycerol-3-phosphate (derived from imported glycerol) and glyceraldehyde-3-phosphate (produced in pentose phosphate pathway), which enter glycolysis downstream of the enzyme. These metabolites might yield lower amounts of ATP though, due to the lower concentration of glycerol in the blood compared to glucose. Buscaglia *et al. *[80] demonstrated that aldolase bridges the actin-myosin motor, which is involved in gliding motility and host cell invasion, and an invasin protein that connects to host cell receptors. Metabolite (and drug) binding to aldolase prevents interactions between aldolase and the invasin protein, which has negative effects on motility and host cell infection. Therefore, the observed reduction of parasitemia upon aldolase inhibition might rather be caused by impaired host cell invasion than metabolic effects.

### Evaluation of predicted drug targets

**Figure 3:**
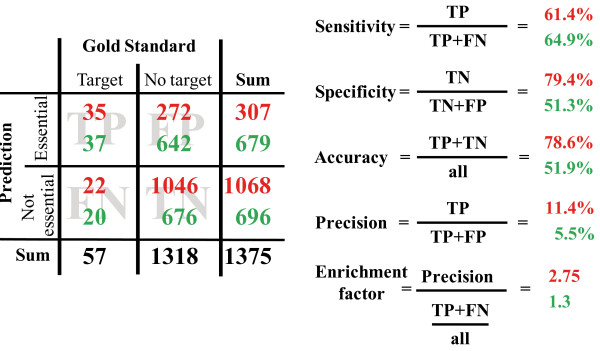
**Evaluation of predicted drug targets. **We conducted FBA based knock-out simulations to uncover reactions within the metabolic network of the parasite that are essential for the production of metabolites assumed to be important for parasite development (see Additional file9). The predicted set of indispensable reactions, which presumably represent good drug targets, was evaluated on the basis of a gold standard set that contains 57 experimentally verified essential enzymes. We determined true positives (TP), false positives (FP), false negatives (FN), and true negatives (TN) and calculated based on these numbers sensitivity, specificity, accuracy and precision of our method as well as the corresponding enrichment factor (red numbers). To compare our method to a previously proposed method for drug target detection, the choke-point analysis, we additionally identified all choke-points within the parasite's metabolic network and calculated the same statistics (green numbers).

Previously, two methods have been presented to predict drug targets in *P. falciparum *[23,24]. Both approaches apply the concept of choke-points, which are reactions that are the only source or sink for at least one metabolite. Such reactions are assumed to be essential due to an accumulation of potentially toxic metabolites or a lack in required substrates upon inhibition. To compare the performance of our method and that of choke-point analysis, all choke-points were identified in the compiled metabolic network and assessed with our gold standard set of drug targets. 679 choke-point reactions were detected in the network, including reactions that are catalyzed by 37 enzymes of our gold standard. Thus, choke-point analysis recovers more true targets than our method. However, due to the large number of reactions predicted as essential by the choke-point analysis, our flux balance approach results in higher values for specificity, accuracy and precision and therefore a better enrichment of true targets (see Figure 3). This means that a choke-point analysis might end up detecting more true targets, but our approach will result in a set of predicted targets with a lower percentage of false positives, which reduces the waste of experimental resources needed to validate the predictions.

To address the question whether some of our predicted targets that are not contained in our gold standard are nevertheless valid targets, the SuperTarget databases [81] was queried with the respective EC numbers to check whether some of these reactions are catalyzed by enzymes that were previously targeted in the course of medical treatments or were shown to have effects on the viability of an organism or pathogenic cell. Of 155 reactions that are assigned to genes and are predicted to be essential but are not covered by the gold standard enzymes, 39 correspond to enzymes that are listed in the SuperTarget database as drug targets.

To avoid unwanted side effects within the human host, it is beneficial to target parasitic enzymes not homologous to human enzymes. Therefore, a BLAST search was conducted on (non-redundant) human protein sequences using the predicted *P. falciparum *protein sequences from PlasmoDB as queries. Analogous to [23] we applied an E-value significance threshold of < 0.075 to determine homologous sequences. Of the 198 reactions that are predicted to be essential and are assigned to genes, 168 are catalyzed by enzymes that are homologous to human enzymes. The gene products catalyzing the remaining 30 reactions represent presumably good drug target candidates. Targets with human homologs may still be interesting targets as long as binding sites for potential inhibitors are sufficiently distinct.

As discussed above, reactions predicted to be essential are not necessarily essential during all time points. Therefore, we considered the stage-specific metabolic fluxes, which were predicted on the basis of gene expression data, to get an idea during which stages reactions are essential. In this context we mapped the consensus reactions calculated in the previous section for each life cycle stage (reactions that carry a non-zero flux in all flux profiles corresponding to the same life cycle stage) onto the set of essential reactions (see Additional file 18). This mapping gives a hint on targets that are present during all time points as opposed to those that are only active during certain stages. Depending on the time point when a reaction of the latter type is essential, drugs designed to inhibit the respective enzyme can either be applied as prophylactic drugs to kill the liver stages of the parasite and prevent disease symptoms or to treat the disease by killing blood stages of the parasite.

In Additional file 18 we ranked predicted drug target reactions that do not correspond to our gold standard set, but are associated with genes, according to a score that represents the sum of weighting factors associated with the following criteria: weighting factor 2 if corresponding genes are not homologous to human genes, weighting factor 1 if the enzymatic function is targeted in any organism (i. e., EC number is listed in the SuperTarget database) and weighting factor 1 if the reaction is active during all parasitic life cycle stages, which makes respective drugs applicable for prophylaxis as well as disease treatment. The 30 top ranking targets are listed in Table 4. Strikingly, among the eight top ranking enzymes/transporters four enzymes and one transporter are assigned to the apicoplast, which harbors prokaryotic biochemical pathways and was therefore proposed as an interesting drug target [82,83]. Three of these top ranking enzymes are involved in the biosynthesis of isopentenyl diphosphate (IPP). Since two enzymes of the IPP biosynthesis pathway (1-deoxy-D-xylulose-5-phosphate reductoisomerase, 2-C-methyl-D-erythritol-2,4-cyclodiphosphate-synthase) have already been suggested as potential targets for antimalarial therapy [84-86], it is likely that other enzymes of this linear pathway have also this potency. *P. falciparum *possesses a pathway to synthesize pyrimidine nucleotides *de novo *of which multiple reactions are predicted to be essential. Some of the corresponding enzymes (carbonic anhydrase, carbamoyl-phosphate synthase, dihydroorotase, dihydroorotate oxidase, orotidine-5'-phosphate decarboxylase) have already been confirmed by experiments to have negative effects on parasite growth upon inhibition [87-98]. These experiments suggest that the parasite is not able to salvage pyrimidine nucleotides and thus other enzymes that catalyze intermediate biosynthesis reaction steps might be likewise essential as proposed by our predictions. The heme biosynthesis pathway has previously been proposed as a target for antimalarial drugs [99-101], as heme is needed as a prosthetic group in proteins such as cytochromes. Heme, which is freed in large amounts during hemoglobin digestion, is presumably unavailable to the parasite outside of the food vacuole [99,102], making *de novo *synthesis essential. This is in accordance with our predictions that classified several enzymes involved in heme biosynthesis to be indispensable. Another pathway that contains reactions predicted to be essential is that of Coenzyme A biosynthesis. It has been demonstrated that the parasite relies on the uptake of pantothenate, a precursor of CoA, and that it is sensitive to pantothenate analogs [103]. Therefore, inhibition of downstream enzymes within this linear pathway might hamper parasite growth as well. Multiple top ranking enzymes are involved in the process of protein biosynthesis (tRNA ligases, deoxyhypusine synthase). The eukaryotic initiation factor, whose activation involves deoxyhypusine synthase, has already been suggested to be the target of a drug with antimalarial effect [104]. Little is known about the translational machinery of *P. falciparum *despite the obvious global effect upon its inhibition. Therefore, an EU funded consortium called MEPHITIS has started very recently to explore the protein synthesis machinery in *Plasmodium *with the goal to design drugs against malaria. Adenylate cyclase obtained among others the highest score by our scoring function. Experimental findings by Ono *et al. *[105] indicate that knock-outs of adenylate cyclase result in a decreased level of cAMP, leading to reduced apical regulated exocytosis and thus impaired infection of host cells. The essentiality of this enzyme is therefore rather related to signaling than to metabolism, but nevertheless represents an effective target. These evidences for the plausibility of our results together with the proven high specificity and accuracy rates for known targets indicate that the predicted set of essential reactions presents a promising starting point for further experimental investigations.

**Table 4:** Top ranking predicted essential reactions

Enzyme name	Compartment	Pathway	EC	Super-Target	Score
adenylate cyclase	apicoplast	Purine metabolism	4.6.1.1	x	4
fumarase	cytosol	Citrate cycle	4.2.1.2	x	4
2-C-methyl-D-erythritol 4-phosphate cytidylyltransferase	apicoplast	Biosynthesis of isopentenyl diphosphate	2.7.7.60	x	4
4-hydroxy-3-methylbut-2-enoyl-diphosphate synthase	apicoplast	Biosynthesis of isopentenyl diphosphate	1.17.4.3		3
CDP-ME kinase	apicoplast	Biosynthesis of isopentenyl diphosphate	2.7.1.148		3
pantetheine-phosphate adenylyltransferase	cytosol	CoA biosynthesis	2.7.7.3		3
pyridoxal 5-phosphate synthase	cytosol	Vitamin B6 metabolism			3
amino acid transporter	Cytosol ↔ apicoplast	Transport			3
geranyl-diphosphate synthase	cytosol	Terpenoid biosynthesis	2.5.1.1	x	2
farnesyl-diphosphate synthase	cytosol	Terpenoid biosynthesis	2.5.1.10	x	2
2-Octaprenylphenol hydroxylase	mitochondrion	Ubiquinone biosynthesis	1.14.13.-	x	2
ubiquinone biosynthesis methyltransferase	mitochondrion	Ubiquinone biosynthesis	2.1.1.-	x	2
uroporphyrinogen decarboxylase	apicoplast	Porphyrin metabolism	4.1.1.37	x	2
coproporphyrinogen oxidase	apicoplast	Porphyrin metabolism	1.3.3.3	x	2
UMP-CMP kinase	apicoplast	Pyrimidine metabolism	2.7.4.14	x	2
aspartate carbamoyltransferase	cytosol	Pyrimidine metabolism	2.1.3.2	x	2
orotate phosphoribosyltransferase	cytosol	Pyrimidine metabolism	2.4.2.10	x	2
cysteine desulfurase	apicoplast/mitochondrion	Fe-S-protein biogenesis	2.8.1.7	x	2
tRNA ligase (Ile, Lys, Met, Trp, Tyr)	cytosol/apicoplast/mitochondrion	Aminoacyl-tRNA biosynthesis	6.1.1.1/6.1.1.2/6.1.1.5/6.1.1.6/6.1.1.10	x	2
glutathione synthase	cytosol	Glutathione metabolism	6.3.2.3	x	2
riboflavin kinase	cytosol	Riboflavin metabolism	2.7.1.26	x	2
glucosamine 6-phosphate synthase	apicoplast	Aminosugars metabolism	2.6.1.16	x	2
mannose-6-phosphate isomerase	cytosol	Fructose and mannose metabolism	5.3.1.8	x	2
cytochrome-c oxidase	mitochondrion	Oxidative phosphorylation	1.9.3.1	x	2
choline-phosphate cytidylyltransferase	cytosol	Glycerophospholipid metabolism	2.7.7.15	x	2
deoxyhypusine synthase	cytosol	Activation of eiF5A	2.5.1.46	x	2

## Discussion

This study has been conducted to explore the capabilities of computational methods to predict metabolic activities during different life cycle stages of the malaria pathogen *P. falciparum*, and furthermore to identify drug targets, given currently available large scale data. For this purpose, a metabolic network was compiled from available resources, which is able to fulfill metabolic functions known from the literature to occur in *P. falciparum*. A common approach to get an insight into the metabolism of an organism is to analyze gene expression data, since this kind of data can be obtained easier than metabolomics data. However, metabolic fluxes do not always correlate well with the transcriptome of a cell [52-55], so that from expression profiles alone no information about flux strength and direction can be gained. Especially in cases where no expression data is available for the pathway of interest, e. g. if no genes are assigned to reactions yet, other methods are needed. The presented flux balance approach facilitates the prediction of stage-specific metabolic flux distributions in accordance with observed gene expression, without requiring detailed kinetic information. In contrast to the original method proposed by Shlomi *et al.*, our approach ensures the production of metabolites assumed to be important for parasite development (phospholipids, amino acids, nucleotides, etc.), yielding presumably more realistic flux predictions. In addition, we simulated host cell conditions for the blood stage parasite by combining the metabolic networks of parasite and erythrocyte and constraining nutrient supply. Predicted nutrient uptake and product secretion were shown to largely agree with experimental findings suggesting that our model of the parasitic metabolism is realistic especially when integrated into its actual environment.

Experimental validation of drug targets by gene knockout techniques is a time consuming issue. Flux balance analysis provides a fast means to reduce the search space. Reactions essential for parasite development are proposed based on their involvement in synthesis processes of indispensable metabolites. Our drug target predictions were evaluated on the basis of a set of known essential enzymes, revealing that the method is able to enrich true targets in the set of predicted targets. The benefits of our FBA approach are good performance (compared to the previously proposed choke-point analysis [23,24]) and short computation times. The latter plays a role especially when the metabolic network of interest is large and beyond the capabilities of topology based methods such as Minimal Cut Sets [106]. We used additional information such as homology to host enzymes and reported negative effects on cell development in other organisms to rank proposed targets. As has been shown for fatty acid synthase [51], which is only essential during late liver stage, the importance of enzymes might vary during different life cycle stages. Therefore, predictions on metabolic fluxes during different time points of parasite development can help to identify reactions that are always active and those that only occur during certain stages, thereby aiding the drug target detection process.

In general, the quality of results derived from computational methods depends heavily on the information used to attain them. For example, about 50% of the predicted genes in *P. falciparum *are not annotated yet, suggesting that our compiled metabolic network is not complete. Furthermore, as pointed out by Ginsburg [107], most of the dababases (BioCyc, KEGG, Reactome) infer metabolic pathways computationally, which results in predictions where actually only one or two enzymes of the pathway are associated with genomic sequences and therefore very likely do not occur in the parasite. To cope with this problem, we removed those pathways that are suggested by [107] to be not present in *P. falciparum*, and in addition, penalized reactions without associated genes in our flux balance calculations. On the one hand, confidence in our drug target predictions increases when such pathways are removed. On the other hand, we might miss interesting targets that are products of yet unannotated genes. For the drug development process, knowledge about the target protein is desired, making predicted target reactions without assigned genes less attractive for further investigation and thus justifies the removal of uncertain pathways.

In addition, one has to keep in mind that the gene expression data sets applied to infer metabolism during the individual life cycle stages are of varying quality. While the blood stage is covered on a close-meshed time scale, the liver stage and especially the mosquito stage are less well analyzed. There's also the fact that the latter stages have partially only been examined in the rodent malaria pathogen which might be different in certain aspects to that of the human malaria pathogen *P. falciparum*. Moreover, the Sacci data set is less expressive than other corresponding data sets, as it is based on a cDNA library rather than microarray analysis and thus captures only part of the true liver stage transcriptome. On top of this, the overall knowledge about the liver and the mosquito stages is still limited compared to the blood stage. As a consequence less information can be included into the models for these stages, making derived predictions less reliable than those for the blood stage.

The accuracy of our predictions is further influenced by the applied criteria that define gene expression as well as posttranscriptional regulation which might lead to discrepancies in transcriptomic and proteomic data. Proteomics and metabolomics data are desirable, as enzyme and metabolite levels give a better picture on the metabolic status of a cell than mRNA levels. Such data is currently not available for as many time points and enzymes/metabolites as it is the case for transcriptomic data. Metabolomics studies have only very recently become available for *P. falciparum*, which measured concentrations for a few metabolites during the blood stage [43,108]. When metabolite concentrations will be available on a larger scale, our method can be extended to incorporate such data. For example, for each detected metabolite one could force at least one reaction producing the metabolite to be active, in order to ensure production of this metabolite. However, metabolite measurements during a single time point do not elucidate whether a metabolite is mainly produced or consumed during the time point of interest and thus such a model extension could be inappropriate. Such information can only be deduced from measurements of two consecutive life cycle time points. Corresponding fluxes could then be fixed in simulations to the difference between the metabolite levels measured during these time points divided by the time difference. Measured metabolite concentrations could also help to attain flux distributions that are consistent with thermodynamics as has been shown in [109]. Predictions could be further improved if more information were available on reaction irreversibilities, transporter capacities, feedback regulations, and nutritional conditions within the different hosts. Last but not least, an accurate definition of target metabolites that are produced during each life cycle stage is needed to obtain good flux predictions for individual stages. Until such data is available, the approach presented here facilitates the generation of working hypotheses that can then be experimentally validated, thereby enhancing the knowledge on the malaria pathogen *P. falciparum*.

## Conclusions

Malaria is still a worldwide health problem and initiatives to fight this disease have to face the increasing spread of resistances to currently available drugs. In this paper, we presented the first compartmentalized metabolic network model of the malaria pathogen *Plasmodium falciparum *and demonstrated how computational methods contribute to malaria research. We introduced a flux balance approach to predict life cycle stage specific metabolism on the basis of gene expression data. Good accordance between predictions and experimental findings was observed for metabolite exchanges between the parasite and its host the human erythrocyte. In order to obtain reliable predictions additional information about the parasite's environment was required, which was implemented by combining the parasite's metabolic network with one of its hosts (erythrocyte) as well as constraints reflecting knowledge about the blood stage. The metabolic network of the hepatocyte, which is currently reconstructed within our group, will soon be available and allow improved predictions in a similar way for liver stage metabolism about which only little is known yet. We additionally conducted knock-out simulations, which revealed a set of indispensable reactions, representing putative drug targets. Evaluation of this set on the basis of experimentally validated essential enzymes confirmed the power of our predictions. Thus, computational methods as presented here have the ability to reduce the search space for novel drug targets in the metabolism of the *P. falciparum*.

## Methods

### Metabolic network compilation

To obtain a network that describes the parasitic metabolism as completely as possible, data from multiple online resources have been integrated in the compilation process. Biochemical reactions were gathered from the Malaria Parasite Metabolic Pathways (MPMP) website (http://sites.huji.ac.il/malaria/, November 2007), the KEGG database (http://www.genome.jp/kegg/, February 2009), the BioCyc database (http://biocyc.org/, release of August 6th 2007 for *P. falciparum *3D7), the Reactome database (http://www.reactome.org/, November 2007), and the BRENDA database (http://www.brenda-enzymes.org/, January 2007). KEGG reactions were considered as present in *P. falciparum*, if genes were assigned via the KEGG Orthology (KO), a classification of orthologous and paralogous groups of genes based on high sequence similarities to genes of known function. Since each resource uses individual identifiers for reactions and metabolites, it was necessary to map the reactions onto each other. The KEGG identifiers were chosen as an overall means of identification. *P. falciparum *reactions suggested by BioCyc and Reactome were mapped to KEGG reactions via annotated genes or EC numbers. In case that way the corresponding KEGG reaction was not found, involved metabolites were mapped to KEGG metabolites either with the help of annotated ChEBI or PubChem identifiers or, if such cross-references were not available, PubChem and ChEBI were queried for matching synonyms. The KEGG metabolite identifiers were then assembled to find the corresponding KEGG reaction. Hagai Gins-burg kindly provided a list of genes and EC numbers corresponding to metabolic pathways known to occur in *P. falciparum*. All KEGG reactions associated with these EC numbers were checked for their presence in any of the pathways depicted at the MPMP website and, where appropriate, added to the parasite's metabolic network. Similarly, all KEGG reactions associated with EC numbers that are annotated as present in *P. falciparum *by the BRENDA database were included in the network. For several reactions listed by the different online resources no matching KEGG reaction could be found. In such cases, the reaction equation translated to respective KEGG metabolites was assigned with a new unique identifier starting with the letter 'Q' followed by five digits in accordance to the KEGG identifiers. Since BioCyc reactions are mostly computationally inferred, we removed reactions that are associated to pathways that are assumed to be not present in *P. falciparum *[107], except for the chorismate biosynthesis pathway.

Each reaction of the resulting metabolic network was assigned to one of the following compartments: cytosol, nucleus, mitochondrion, apicoplast, endoplasmic reticulum, or food vacuole. The default compartment for all reactions is the cytosol. This assignment was overruled if the corresponding gene is annotated with Gene Ontology terms [110] within the PlasmoDB database [111] that refer to another compartment. Compartment reassignment was done as well for reactions either assigned by the MPMP website to a different compartment or when corresponding genes contain signal sequences for the apicoplast or mitochondrion. Information on transporters transferring metabolites between the different compartments as well as between the host and the parasite, were mostly obtained from the MPMP website, and in addition, from the Transport Classification database http://www.tcdb.org/, BioCyc, and Reactome database. The metabolic networks of *P. falciparum *and the human erythrocyte are both available as SBML files (Additional files 1 and 2).

### Consistency checks

In a first step highly specific metabolites such as beta-D-Glucose were replaced by more general metabolites (D-Glucose), using a manually compiled list of substitutions. Moreover, if the same metabolite occurs with different IDs in reactions, one was chosen to replace alternative IDs. Reactions containing generic terms like 'acceptor' or 'fatty acid' were either removed, replaced by more specific reactions or, if the generic metabolite matched multiple specific metabolites, a pseudo-reaction was added converting the metabolite in more specific ones (e. g. fatty acid → hexadecanoate + (9Z)-Hexadecenoic-acid + octadecanoic-acid + (9Z)-Octadecenoic-acid + (9Z,12Z)-Octadecadienoic-acid). Furthermore, generic reactions (A + n B → n C + D) were removed. Finally, reaction duplets were deleted that were already present in the original data or were generated by the consistency procedure.

### Flux balance analysis

Flux balance analysis (FBA) is a commonly used method to investigate the metabolic capabilities of cellular systems, since it allows to estimate unknown fluxes in metabolic networks with respect to cellular constraints [10,19,112-115]. Among others, FBA has been used for metabolic engineering studies [116,117], drug target prediction [118-120] as well as growth prediction on different media [121,122]. In contrast to kinetic modeling no detailed rate equations are required, predestining the method for large metabolic networks, which lack full kinetic characterization of all enzymes. Prerequisite for application of this method is the stoichiometric matrix *S*, which describes the number of metabolite molecules consumed and produced by each reaction of the metabolic network, as well as knowledge about the cellular objective. The basis of FBA is the flux conservation principle, which assumes the system to be at quasi steady state with metabolite concentrations [*X*] and reaction fluxes *v *being constant, since bio-chemical reactions are typically much faster than changes in the phenotype of a cell during growth or differentiation:

$$\frac{d\left[X\right]}{dt}=Sv=0$$

The solution space of the resultant linear equation system is a high dimensional flux cone, containing all flux configurations that are feasible at steady state. Constraints derived from thermodynamics and cellular environment conditions further restrict the solution space. Organisms are thought to be optimized in the course of evolution. Therefore, a biologically meaningful objective function, which is a linear function of fluxes, is applied to the set of possible solutions to identify a flux distribution that is optimal with respect to the cellular objective. Maximization of biomass is one such target function that is assumed to be suitable especially for microorganisms [123]. For eukaryotic cells such as hepatocytes, for which reproduction is not the primary goal, cellular maintenance at minimal efforts has been proposed as an alternative objective function [124]. Altogether this yields the following formalism:

$$\begin{array}{cc} \min\;\;/\underset{v}{\max} & c^{T}v\;\;(\text{objective function})\\ \text{s}\text{. t}\text{.}: & Sv=0\;\;(\text{steady state assumption})\\ & v_{\min,i}<v_{i}<v_{\max,i}\;(\text{constraints}) \end{array}\;$$

where *S *is the stoichiometric matrix of the cellular system, *v *are internal fluxes as well as exchange fluxes, and *v_min _*and *v_max _*are the lower and upper bounds on fluxes.

### Evaluating the expression status of genes

The expression status was determined for each gene of every expression sample. For the Le Roch data set the same parameter cutoffs were applied as mentioned in [6] (thermocycling treatments data): expression level *E *> 10 and likelihood of gene absence calculated by the match-only integral distribution algorithm *logP *< -0.5. Genes covered by the Bozdech and Tarun data sets were considered to be expressed if the corresponding signal-to-noise ratio (*s*2*n *= (*mean*(Foreground signal) - *mean*(Background signal))/*std*(Background signal), with Foreground signal being the signal resulting from specific hybridization and Background signal being the noise caused by non-specific binding), is greater or equal to a threshold. Signal-to-noise ratios were extracted from the raw data, averaged over all replicates and normalized using quantile normalization [125]. Applying a too low or too high threshold results in almost all genes being expressed or silenced, respectively. Therefore, the threshold *t *was chosen for the Bozdech data set in such a way that the average Euclidean distance between the binary expression values (*expr *(*g *| *t*) = **0: **gene *g *is silent given threshold *t*, *expr *(*g *| *t*) = **1: **gene *g *is expressed given threshold *t*) is maximal for non-consecutive pairs of samples of the same data set and minimal for consecutive pairs:

$$\begin{array}{c} \max\arg_{t}\frac{\sum_{i=1}^{N}\sum_{j=i+2}^{N}\sqrt{\sum_{k=1}^{M}{\left[expr\left(i_{k}|t\right)-expr\left(j_{k}|t\right)\right]}^{2}}}{\sum_{i=1}^{N}\sum_{j=i+2}^{N}1}\\ -\frac{\sum_{i=1}^{N}\sum_{j=i+1}^{i+1}\sqrt{\sum_{k=1}^{M}{\left[expr\left(i_{k}|t\right)-expr\left(j_{k}|t\right)\right]}^{2}}}{\sum_{i=1}^{N}\sum_{j=i+1}^{i+1}1} \end{array}$$

where *N *is the number of gene expression samples, *M *is the number of genes and *i_k _*is the *k^th ^*gene in sample *i*.

A threshold of 17 turned out to be optimal. This approach could not be applied to the Tarun set, since the time span between extractions of samples is much larger. Instead, the signal-to-noise ratio threshold was set to 25 which yielded largest similarity between Tarun samples and corresponding Le Roch samples. As the Sacci data set is a cDNA library that captures mRNA present at a certain time point, all genes of the library were accounted as expressed. For the Daily samples a gene expression signal cutoff of 139 yielded largest similarity between samples of cluster 2 (active growth) and ring stage expression samples of the Le Roch set and was therefore used to determine expression in Daily samples.

### Calculating flux distributions consistent with gene expression data

Flux distributions were predicted for the different life cycle stages of the malaria pathogen with an extended version of the method proposed by Shlomi *et al. *[25]. This method is a flux balance approach with additional binary variables for reactions with associated gene expression data. Constraints force reactions to have a non-zero (zero) flux value if the respective gene is expressed (not expressed) and the corresponding binary variable (*y*_1*p *_(forward direction), *y*_1*n *_(backward direction), *y*_0 _(no flux)) is set to 1. The sum of these binary variables composes the objective function that is sought to be maximized to obtain flux distributions that are consistent with corresponding expression profiles. However, the Shlomi method has several limitations: synthesis of metabolites needed for growth is not guaranteed, reactions of low confidence are treated the same as those with annotated genes, in order to maximize the objective function all reactions catalyzed by the same gene product are sought to be active simultaneously in case the gene is expressed and furthermore no limits are set on available nutrients. To cope with these issues we extended the method as follows. (a) Additional constraints force the network to produce a set of target metabolites that are assumed to be essential during the life cycle stage of interest (see Additional file 9). (b) For all reactions that have no associated gene, boolean variables (*y*_2,*i*_) and constraints were introduced that keep these reactions inactive if the respective variable is set to 1. (c) For each expressed gene a boolean variable (*y*_3,*i*_) and constraints were added, making sure that at least one of the reactions associated to an expressed gene has to carry a non-zero flux in case the respective variable is set to 1. Replacing variables *y*_1*p *_and *y*_1*n *_in the objective function with this variable type avoids that all reactions catalyzed by the same expressed enzyme are sought to be active at the same time to further optimize the objective function value. (d) With respect to the restrained access of the parasite to nutrients of the host the network is forced to fulfill its tasks with a minimum amount of externally supplied metabolites by subtracting the absolute flux values through the parasite's plasma membrane transporters from the objective function value. (e) To simulate the parasites environment the uptake of certain metabolites was constrained in accordance with knowledge about the blood stage. The corresponding mixed integer linear program reads as follows:

(1) $$\begin{array}{c} \underset{y_{0},y_{2},y_{3}}{\underset{v^{+},v^{-},}{\max}}\;\sum_{i\in R_{NE}}\alpha y_{0,i}+\sum_{i\in R_{NG}}\beta y_{2,i}\\ \;\;\;\;\;+\sum_{i\in G_{E}}\text{γ}y_{3,i}-\sum_{i\in T}\delta v_{i}^{+} \end{array}$$

(2) s. t.: Sv=0

(3) $$v_{\min,i}\leq v\leq v_{\max,i}$$

(4) $$v_{i}\geq 1,\;\text{ for all target fluxes}$$

(5) $$\begin{array}{l} v_{i}+y_{1p,i}(v_{\min,i}-\epsilon )\geq v_{\min,i},\\ \;\forall i\in R_{E} \end{array}$$

(6) $$\begin{array}{l} v_{i}+y_{1n,i}(v_{\max,i}+\epsilon )\leq v_{\max,i},\\ \;\forall i\in R_{E} \end{array}$$

(7) $$\begin{array}{l} v_{\min,i}\left(1-y_{0}\right)\leq v_{i}\leq v_{\max,i}\left(1-y_{0}\right),\\ \;\forall i\in R_{NE} \end{array}$$

(8) $$\begin{array}{l} v_{\min,i}\left(1-y_{2}\right)\leq v_{i}\leq v_{\max,i}\left(1-y_{2}\right),\\ \;\forall i\in R_{NG} \end{array}$$

(9) $$y_{3,i}-y_{1p,r(i)}\geq 0,\;\forall i\in G_{E}$$

(10) $$y_{3,i}-y_{1n,r(i)}\geq 0,\;\forall i\in G_{E}$$

(11) $$\begin{array}{l} y_{3,i}\leq \sum y_{1p,r(i)}+\sum y_{1n,r(i)},\\ \;\forall i\in G_{E} \end{array}$$

(12) $$v=v^{+}-v^{-}$$

(13) $$y_{0},y_{1p},y_{1n},y_{2},y_{3}\in \left[0,1\right]$$

(14) $$v^{+},v^{-}\geq 0$$

Within the solution space, which is defined by equations and inequalities (2)-(14), a flux distribution is sought that maximizes the objective function described by (1). *α *denotes the reward for setting the fluxes of reactions to zero that correspond to unexpressed genes (*R_NE_*), *β *represents the reward for assigning a zero flux to such reactions that are not associated with any gene (*R_NG_*), γ denotes the reward for having a non-zero flux through any reaction assigned to an expressed gene (*G_E_*), while *δ *is the penalty for forward fluxes *v*^+ ^through parasitic plasma membrane transporters (*T*) importing external metabolites. In our calculations the variables were set as follows: *α *= 2, *β *= 0.1, γ = 1, and *δ *= 0.5. Inequalities (5)-(7) are the same as in [25] and (8) is analogous to (7) to obtain a zero flux through reactions without associated gene. The subsequent inequalities (9)-(11) ensure that if a binary variable of type *y*_3 _is set to 1, at least one of the corresponding variables of type *y*_1 _have to equal 1 as well. Matching variables *y*_1 _and *y*_3 _are identified via function *r*(*i*) that maps a gene onto a set of reactions that are catalyzed by the gene product. Biomass production is ensured by (4), which requires e fluxes of at least one molecule of the respective target metabolites through system boundaries.

### Detection of essential reactions

FBA methods can be used to determine reactions of a metabolic network that are essential for the synthesis of certain target metabolites such as biomass precursors by simulating the effects of knocked-out genes [118-120,126,127]. The linear program underlying the knock-out simulations of this study consists of equations derived from the steady state assumption as well as thermodynamic constraints and constraints demanding the production of metabolites assumed to be essential during any time point of parasite development (equation (2) and inequalities (3) and (4) of previous section). In addition, fluxes through a reaction of interest are fixed to zero, mimicking the knock-out of the corresponding gene. For each reaction of the metabolic network of *P. falciparum *such a linear program was formulated. In case the solution space of the problem was empty, the knocked-out reaction was considered to be indispensable and thus a putative drug target, since no path exists converting external nutrients into all target metabolites, which presumably results in impaired parsite development. In contrast to the previously conducted flux balance analysis no gene expression is considered for the identification of essential reactions.

## List of abbreviations used

FBA: flux balance analysis; IDC: intraerythrocytic developmental cycle

## Authors' contributions

CH developed the original idea, carried out all computational analyses and drafted the manuscript. AH provided tools for the knock-out simulations and together with HGH and SB participated in the design and evaluation of the analyses. All authors contributed to and approved the final manuscript.

## Supplementary Material

Additional file 1 **Assembled metabolic network of P. falciparum in SBML format**. Click here for file

Additional file 2 **Assembled metabolic network of the human erythrocyte in SBML format**. Click here for file

Additional file 3 **Gene expression samples**. In order to calculate a stage-specific flux distributions our flux balance approach requires a gene expression profile of the stage of interest. We obtained gene expression samples from different publications, including Bozdech *et al.*, Le Roch *et al.*, Sacci *et al.*, and Tarun *et al.* Click here for file

Additional file 4 **Normalized Hamming distance matrix for gene expression samples**. In order to compare the gene expression profiles of the different time points normalized Hamming distances (see text for formula) have been calculated as described in the text for each pair of gene expression samples. The darker the color of a matrix entry, the lower is the corresponding Hamming distance. Sample labels are composed of the sample abbreviation and the number of expressed genes. Click here for file

Additional file 5 **Bozdech gene expression samples mapped onto metabolic pathways**. Mapping gene expression data onto metabolic networks may uncover active pathways for each stage and metabolic differences between the individual life cycle stages. For this purpose, we calculated the ratio of expressed genes per KEGG pathway (# expressed genes/# genes with available expression data for pathway) for each Bozdech gene expression sample. The darker the color of a matrix entry, the lower is the ratio. Clusters of pathways with similar patterns of expressed genes during the individual life cycle time points were calculated with the built-in function hclust ('average' method) of the statistics software **R **(colored bars). Click here for file

Additional file 6 **Le Roch gene expression samples mapped onto metabolic pathways**. See caption of Additional file 5. Click here for file

Additional file 7 **Tarun gene expression samples mapped onto metabolic pathways**. See caption of Additional file 5. Click here for file

Additional file 8 **Daily gene expression samples mapped onto metabolic pathways**. See caption of Additional file 5. Click here for file

Additional file 9 **Metabolites essential for parasite development**. Metabolites listed in this table are assumed to be essential during those developmental stages of the parasite that are specified in the third column. Some entries represent pseudo-metabolites that do not correspond to actual metabolites. These have been added as products in the equations of certain important reactions such as glutathione reductase. Requiring the production of these pseudo-metabolite ensures that the respective reaction is active. Click here for file

Additional file 10 **Normalized Hamming distance matrix for calculated flux distributions**. Flux distributions have been predicted with our flux balance approach (see Figure 1) for each time point of the parasite's life cycle for which a gene expression profile exists. Simulations were conducted considering only the metabolic network of the parasite without any further constraints reflecting the parasite's environment and without considering the expression status of genes during preceding time points. In order to compare the individual flux distributions normalized Hamming distances (see text for formula) have been determined for all pairs of flux distributions. The darker the color of a matrix entry, the lower is the corresponding Hamming distance. Click here for file

Additional file 11 **Predicted host parasite metabolite exchanges**. Flux distributions have been predicted with our flux balance approach (see Figure 1) for each time point of the parasite's life cycle for which a gene expression profile exists. Simulations were conducted considering only the metabolic network of the parasite without any further constraints reflecting the parasite's environment and without considering the expression status of genes during preceding time points. Resulting metabolite exchanges between host and parasite are depicted in this figure. Red matrix entries represent metabolites that are predicted to be imported into the parasite while green matrix entries represent metabolites secreted into the host compartment. Click here for file

Additional file 12 **Normalized Hamming distance matrix for calculated flux distributions using improved approach**. Flux distributions have been predicted with our improved flux balance approach (see Figure 1) for each time point of the intraerythrocytic developmental cycle for which a gene expression profile exists. Simulations were conducted on the basis of the combined metabolic network of parasite and host and additional constraints reflecting knowledge about the blood stage. Furthermore, the expression status of genes during preceding time points was considered for the flux calculations. In order to compare the individual flux distributions normalized Hamming distances (see text for formula) have been determined for all pairs of flux distributions. The darker the color of a matrix entry, the lower is the corresponding Hamming distance. Click here for file

Additional file 13 **Reaction distribution among stage-specific fluxes**. Flux distributions have been predicted with our improved flux balance approach (see Figure 1) for each time point of the intraerythrocytic developmental cycle for which a gene expression profile exists. Simulations were conducted on the basis of the combined metabolic network of parasite and host and additional constraints reflecting knowledge about the blood stage. Furthermore, the expression status of genes during preceding time points was considered for the flux calculations. Subsequently, it was counted in how many of these stage-specific flux distributions a particular reaction was carrying a non-zero flux. The resulting histogram is shown here. The x-axis gives the number of flux distributions within which a reaction carries a non-zero flux and the y-axis indicates the frequency. In other words, the left most bar of the histogram represents the number of reactions that exclusively occur in a single flux distribution and are therefore very stage-specific, while the right most bar represents the number of reactions that are present in all (96) flux distributions related to the blood stage. Click here for file

Additional file 14 **Predicted metabolic fluxes consistent with Bozdech gene expression data mapped onto metabolic pathways**. Flux distributions have been predicted with our improved flux balance approach (see Figure 1) for each time point of the intraerythrocytic developmental cycle for which a gene expression profile exists. Simulations were conducted on the basis of the combined metabolic network of parasite and host and additional constraints reflecting knowledge about the blood stage. Furthermore, the expression status of genes during preceding time points was considered for the flux calculations. In order to explore the predicted flux distributions on the level of metabolic pathways, we mapped the flux profiles onto KEGG pathways and counted active reactions, to assess whether a pathway is active or not and wether there are changes during the IDC. The darker the color of a matrix entry the fewer reactions of the corresponding pathway are active. Click here for file

Additional file 15 **Predicted metabolic fluxes consistent with Le Roch gene expression data mapped onto metabolic pathways**. Same as Additional file 14 but fluxes were calculated using Le Roch gene expression data. Click here for file

Additional file 16 **Predicted metabolic fluxes consistent with Daily gene expression data mapped onto metabolic pathways**. Same as Additional file 14 but fluxes were calculated using Daily gene expression data. Click here for file

Additional file 17 **Overview of pathway specific consensus reactions for different time points of intraerythrocytic cycle**. Flux distributions have been predicted with our improved flux balance approach (see Figure 1) for each time point of the intraerythrocytic developmental cycle for which a gene expression profile exists. Simulations were conducted on the basis of the combined metabolic network of parasite and host and additional constraints reflecting knowledge about the blood stage. Furthermore, the expression status of genes during preceding time points was considered for the flux calculations. Consensus reactions, which are reactions that are predicted to be active for all gene expression samples covering the same stage, were determined. These reactions are more likely to actually occur during a certain stage, since they are derived from different data samples. In order to identify consensus reactions we grouped all calculated flux profiles corresponding to the blood stage into seven sets (eRing, lRing, eTropho, lTropho, eSchiz, lSchiz, Mero; see Additional file 3) with respect to represented stages and determined those reactions that carry a non-zero flux in all flux profiles of the same set. For each blood stage and each metabolic pathway the fraction of consensus reactions per total number of pathway reactions was computed to uncover the distribution of consensus reactions among pathways. To get an impression of how many consensus reactions are shared between the different stages, this fraction was also computed for those consensus reactions that two sets have in common. The darker the color of a matrix entry the lower is the percentage of consensus reactions. Click here for file

Additional file 18 **Ranked predicted essential reactions**. Knock-outs were simulated with a simple FBA approach neglecting gene expression information. No assumptions were made about the cellular environment and nutrient uptake was not restricted, since such information is not available for all stages. Successively reactions were constrained to carry no flux, while the network was forced to produce all metabolites that are assumed to be essential during any developmental stage (all metabolites listed in Additional file 9). If no solution could be found to this problem the reaction was assumed to be essential. The resulting set of indispensable reactions, which are assigned to genes and not covered by our gold standard set of experimentally validated essential enzymes, is listed here. Reactions are ranked according to a score that is derived as follows: two points if corresponding genes are not homologous to human genes, an additional point if the reaction is targeted in any other organism (according to the SuperTarget database), and another additional point if the reaction is active during all parasitic life cycle stages, which makes respective drugs applicable for prophylaxis as well as disease treatment. Click here for file
