# Supplementary figures and images for: Antimalarial drug targets in Plasmodium falciparum predicted by stage-specific metabolic network analysis
Source: BMC Syst Biol. 2010 Aug 31;4:120. doi: 10.1186/1752-0509-4-120 (PMC2941759; doi:10.1186/1752-0509-4-120)

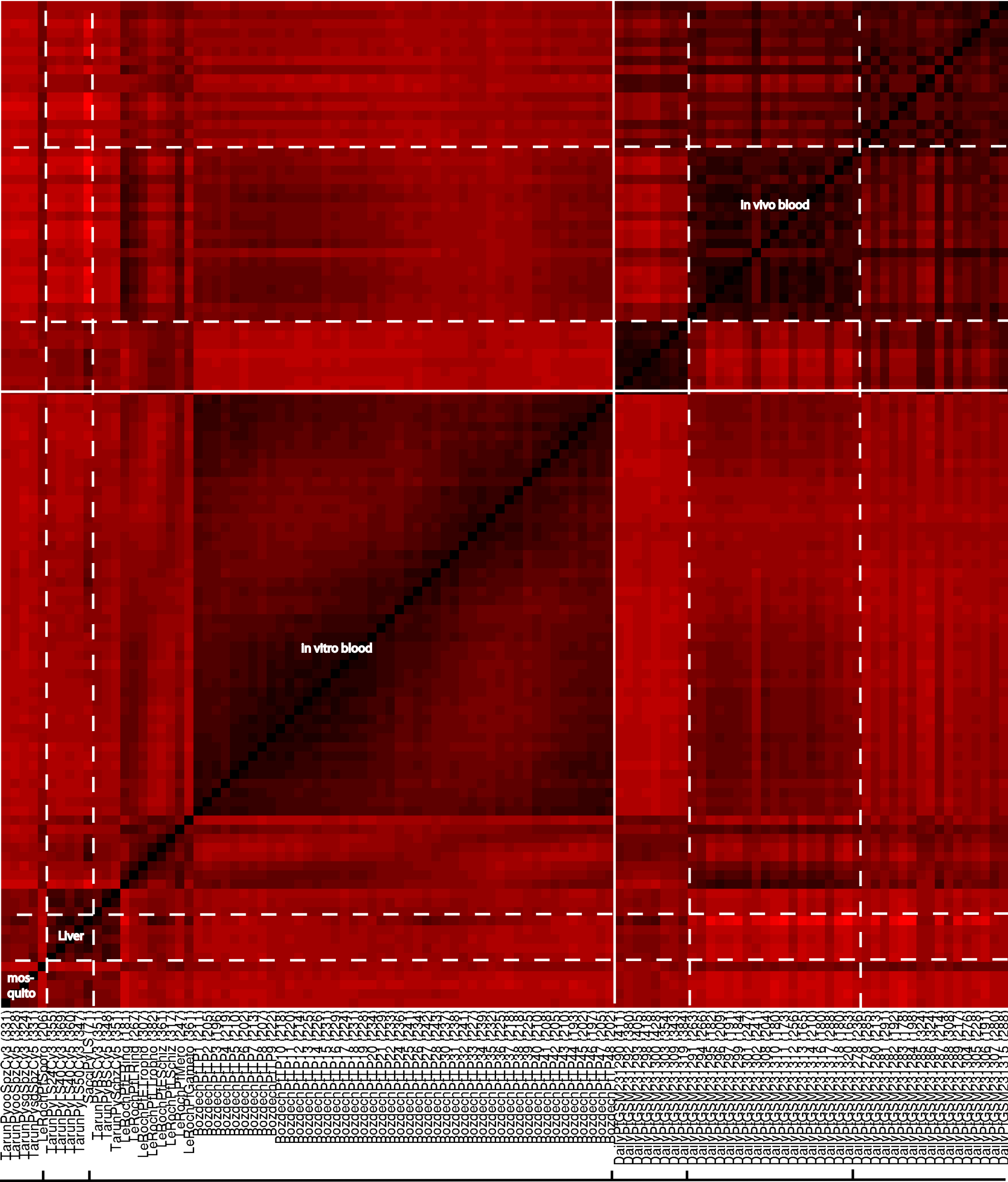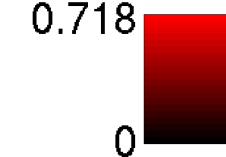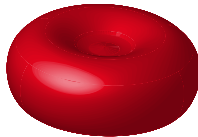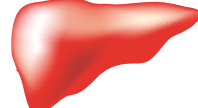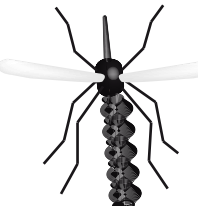

Tarun & Le Roch  
Tarun & Sacci  
Tarun & Le Roch & Bozdech  
Cluster 1  
Cluster 2  
Cluster 3  
Daily

Supplement: Additional file 4 — Normalized Hamming distance matrix for gene expression samples. In order to compare the gene expression profiles of the different time points normalized Hamming distances (see text for formula) have been calculated as described in the text for each pair of gene expression samples. The darker the color of a matrix entry, the lower is the corresponding Hamming distance. Sample labels are composed of the sample abbreviation and the number of expressed genes. [file 1752-0509-4-120-S4.PDF]

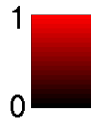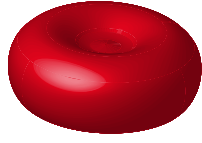

Supplement: Additional file 5 — Bozdech gene expression samples mapped onto metabolic pathways. Mapping gene expression data onto metabolic networks may uncover active pathways for each stage and metabolic differences between the individual life cycle stages. For this purpose, we calculated the ratio of expressed genes per KEGG pathway (# expressed genes/# genes with available expression data for pathway) for each Bozdech gene expression sample. The darker the color of a matrix entry, the lower is the ratio. Clusters of pathways with similar patterns of expressed genes during the individual life cycle time points were calculated with the built-in function hclust ('average' method) of the statistics software R (colored bars). [file 1752-0509-4-120-S5.PDF]

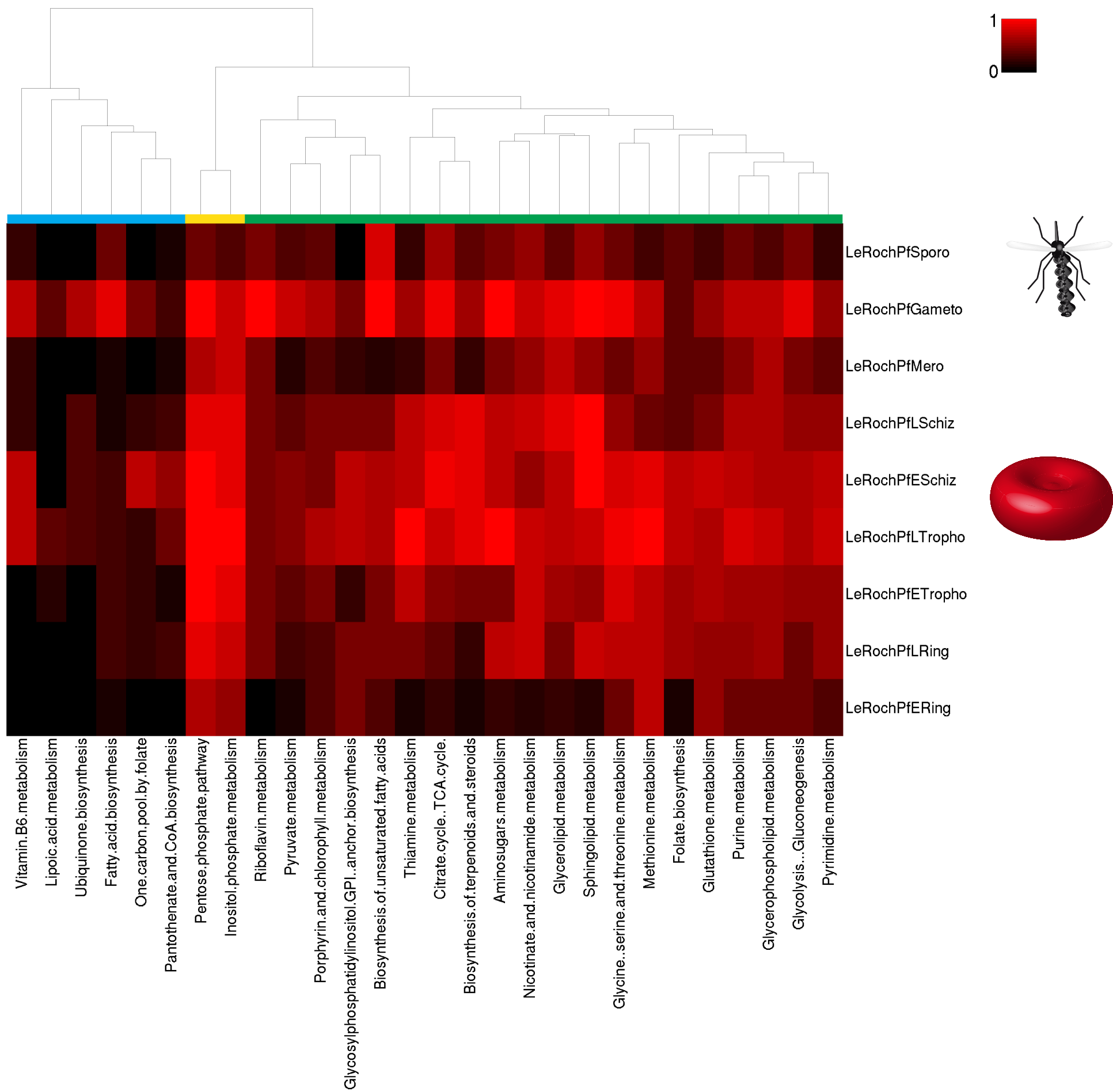

Supplement: Additional file 6 — Le Roch gene expression samples mapped onto metabolic pathways. See caption of Additional file 5. [file 1752-0509-4-120-S6.PDF]

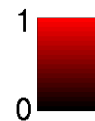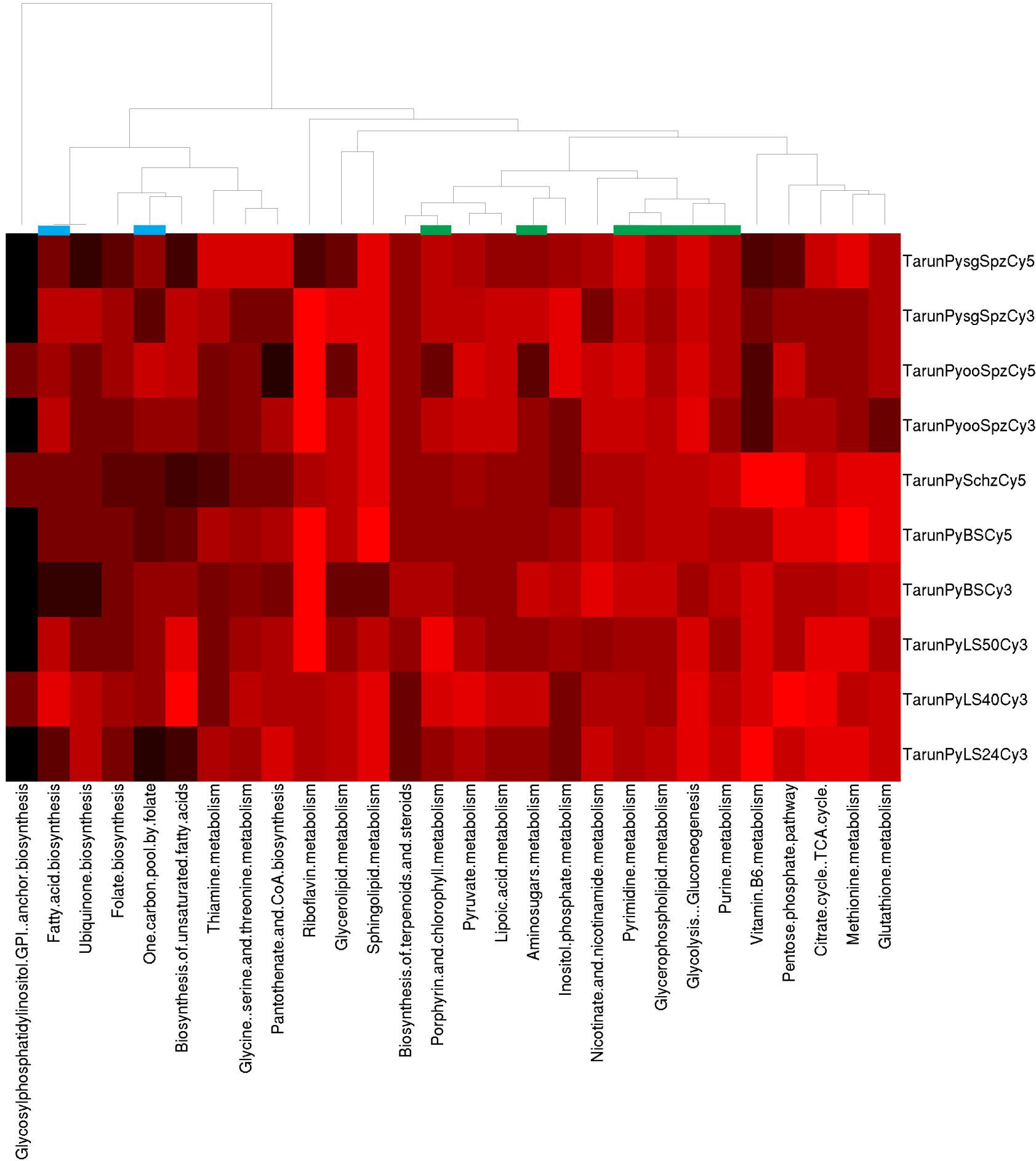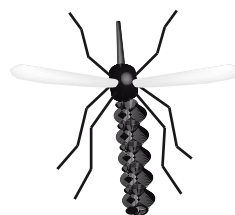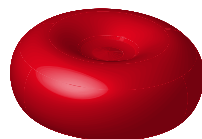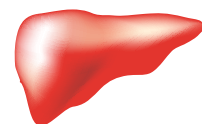

Supplement: Additional file 7 — Tarun gene expression samples mapped onto metabolic pathways. See caption of Additional file 5. [file 1752-0509-4-120-S7.PDF]

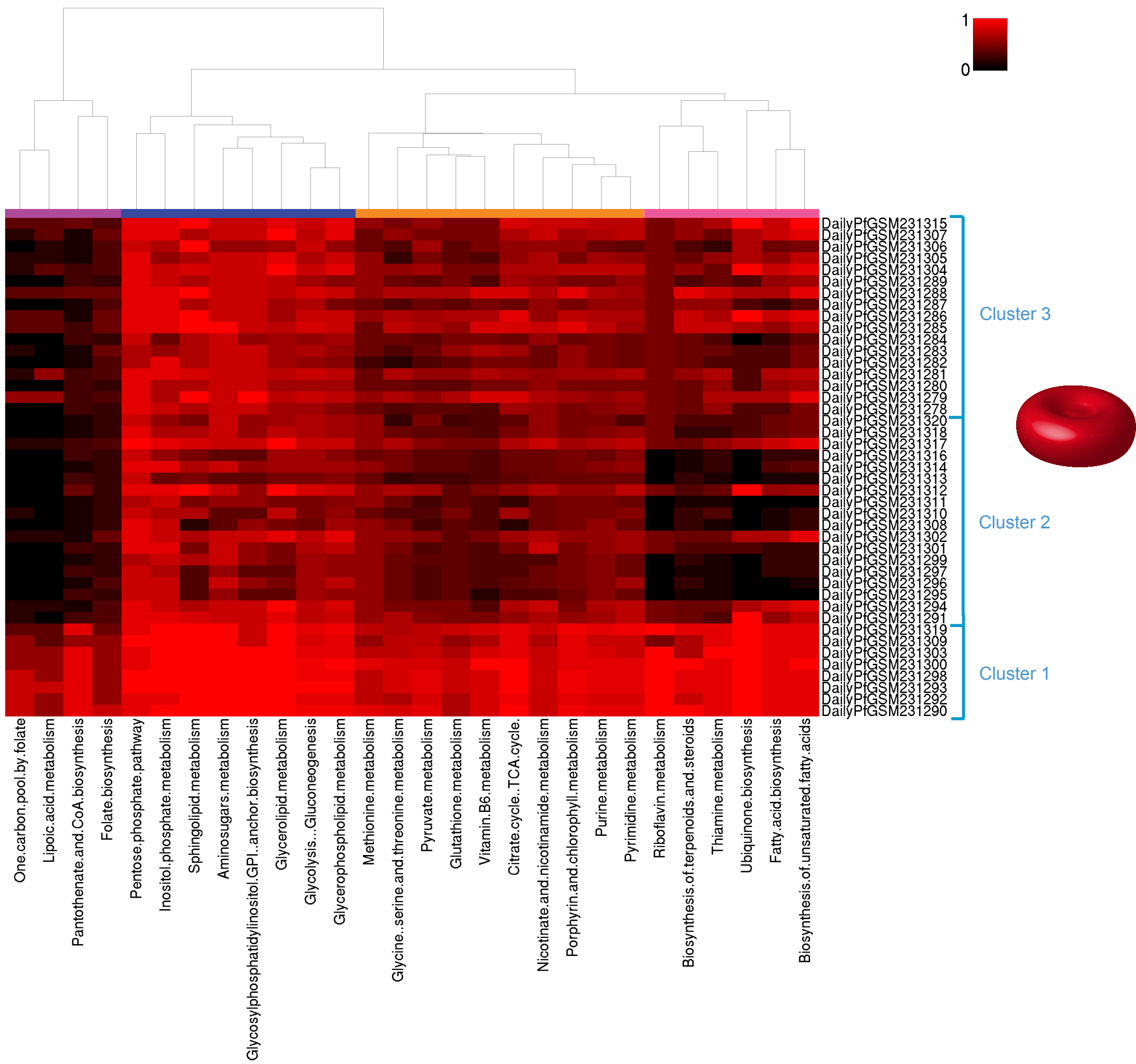

Supplement: Additional file 8 — Daily gene expression samples mapped onto metabolic pathways. See caption of Additional file 5. [file 1752-0509-4-120-S8.PDF]

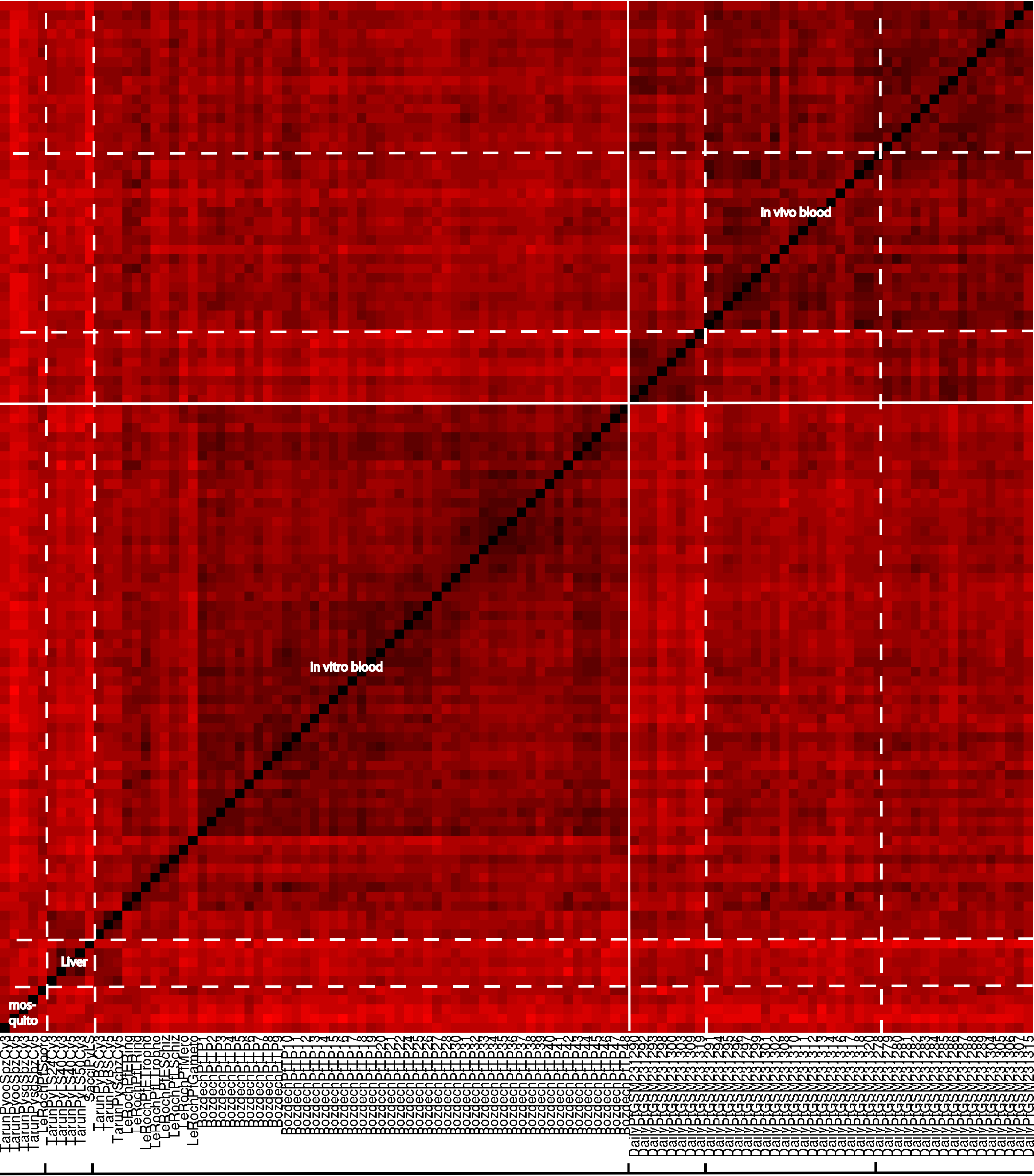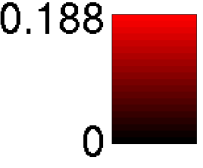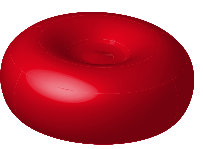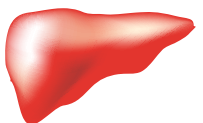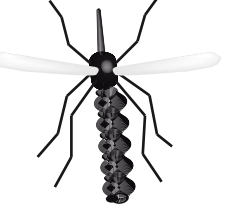

Tarun & Le Roch  
Tarun & Sacci

Tarun & Le Roch & Bozdech

Cluster 1

Cluster 2

Daily

Cluster 3

Supplement: Additional file 10 — Normalized Hamming distance matrix for calculated flux distributions. Flux distributions have been predicted with our flux balance approach (see Figure 1) for each time point of the parasite's life cycle for which a gene expression profile exists. Simulations were conducted considering only the metabolic network of the parasite without any further constraints reflecting the parasite's environment and without considering the expression status of genes during preceding time points. In order to compare the individual flux distributions normalized Hamming distances (see text for formula) have been determined for all pairs of flux distributions. The darker the color of a matrix entry, the lower is the corresponding Hamming distance. [file 1752-0509-4-120-S10.PDF]

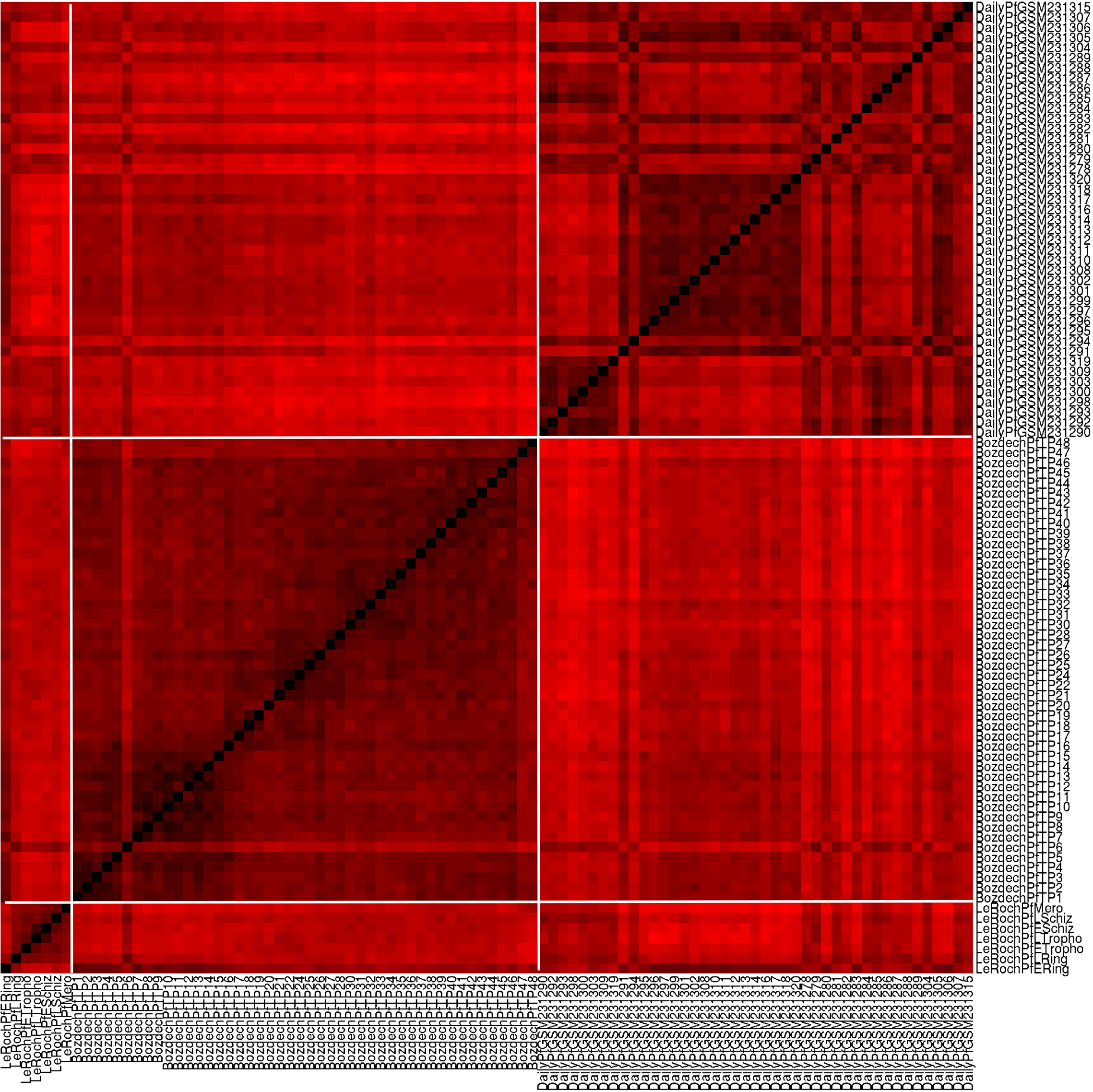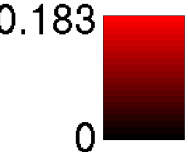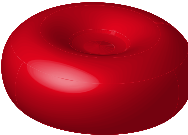

Le Roch                      Bozdech                      Cluster 1                      Cluster 2                      Cluster 3

Daily

Supplement: Additional file 12 — Normalized Hamming distance matrix for calculated flux distributions using improved approach. Flux distributions have been predicted with our improved flux balance approach (see Figure 1) for each time point of the intraerythrocytic developmental cycle for which a gene expression profile exists. Simulations were conducted on the basis of the combined metabolic network of parasite and host and additional constraints reflecting knowledge about the blood stage. Furthermore, the expression status of genes during preceding time points was considered for the flux calculations. In order to compare the individual flux distributions normalized Hamming distances (see text for formula) have been determined for all pairs of flux distributions. The darker the color of a matrix entry, the lower is the corresponding Hamming distance. [file 1752-0509-4-120-S12.PDF]

## Active reactions in different flux distributions

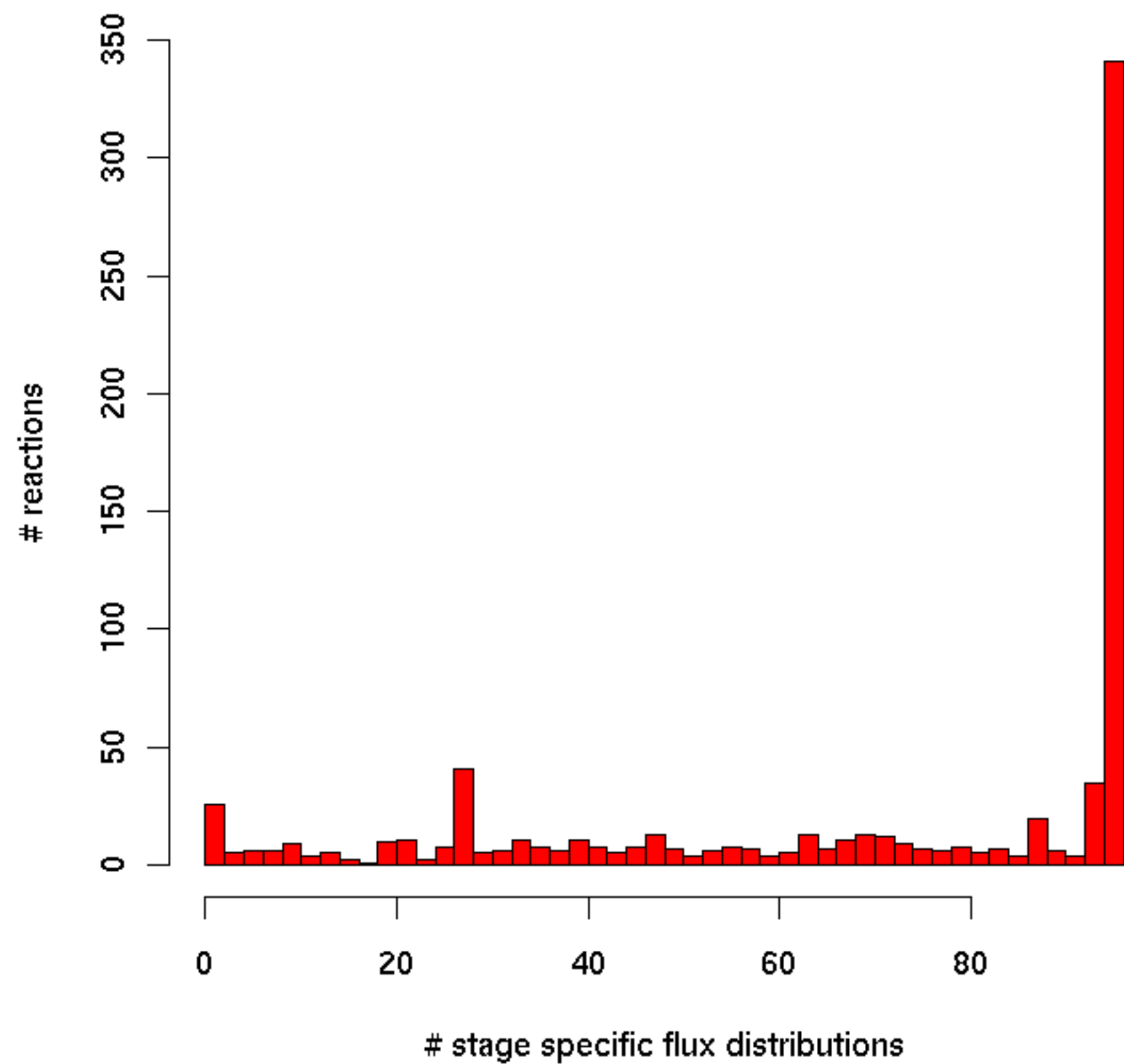

Supplement: Additional file 13 — Reaction distribution among stage-specific fluxes. Flux distributions have been predicted with our improved flux balance approach (see Figure 1) for each time point of the intraerythrocytic developmental cycle for which a gene expression profile exists. Simulations were conducted on the basis of the combined metabolic network of parasite and host and additional constraints reflecting knowledge about the blood stage. Furthermore, the expression status of genes during preceding time points was considered for the flux calculations. Subsequently, it was counted in how many of these stage-specific flux distributions a particular reaction was carrying a non-zero flux. The resulting histogram is shown here. The x-axis gives the number of flux distributions within which a reaction carries a non-zero flux and the y-axis indicates the frequency. In other words, the left most bar of the histogram represents the number of reactions that exclusively occur in a single flux distribution and are therefore very stage-specific, while the right most bar represents the number of reactions that are present in all (96) flux distributions related to the blood stage. [file 1752-0509-4-120-S13.PDF]

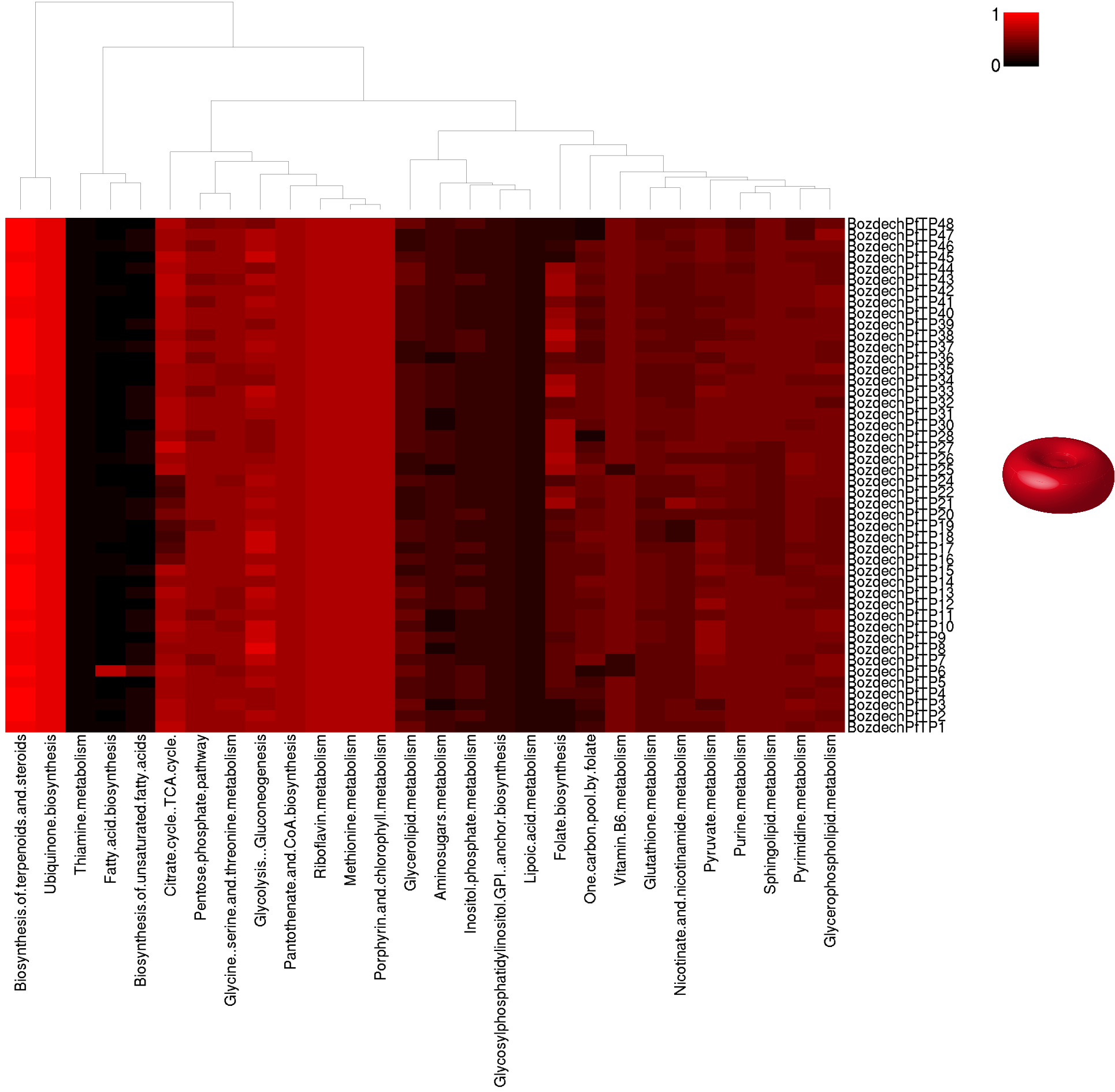

Supplement: Additional file 14 — Predicted metabolic fluxes consistent with Bozdech gene expression data mapped onto metabolic pathways. Flux distributions have been predicted with our improved flux balance approach (see Figure 1) for each time point of the intraerythrocytic developmental cycle for which a gene expression profile exists. Simulations were conducted on the basis of the combined metabolic network of parasite and host and additional constraints reflecting knowledge about the blood stage. Furthermore, the expression status of genes during preceding time points was considered for the flux calculations. In order to explore the predicted flux distributions on the level of metabolic pathways, we mapped the flux profiles onto KEGG pathways and counted active reactions, to assess whether a pathway is active or not and wether there are changes during the IDC. The darker the color of a matrix entry the fewer reactions of the corresponding pathway are active. [file 1752-0509-4-120-S14.PDF]

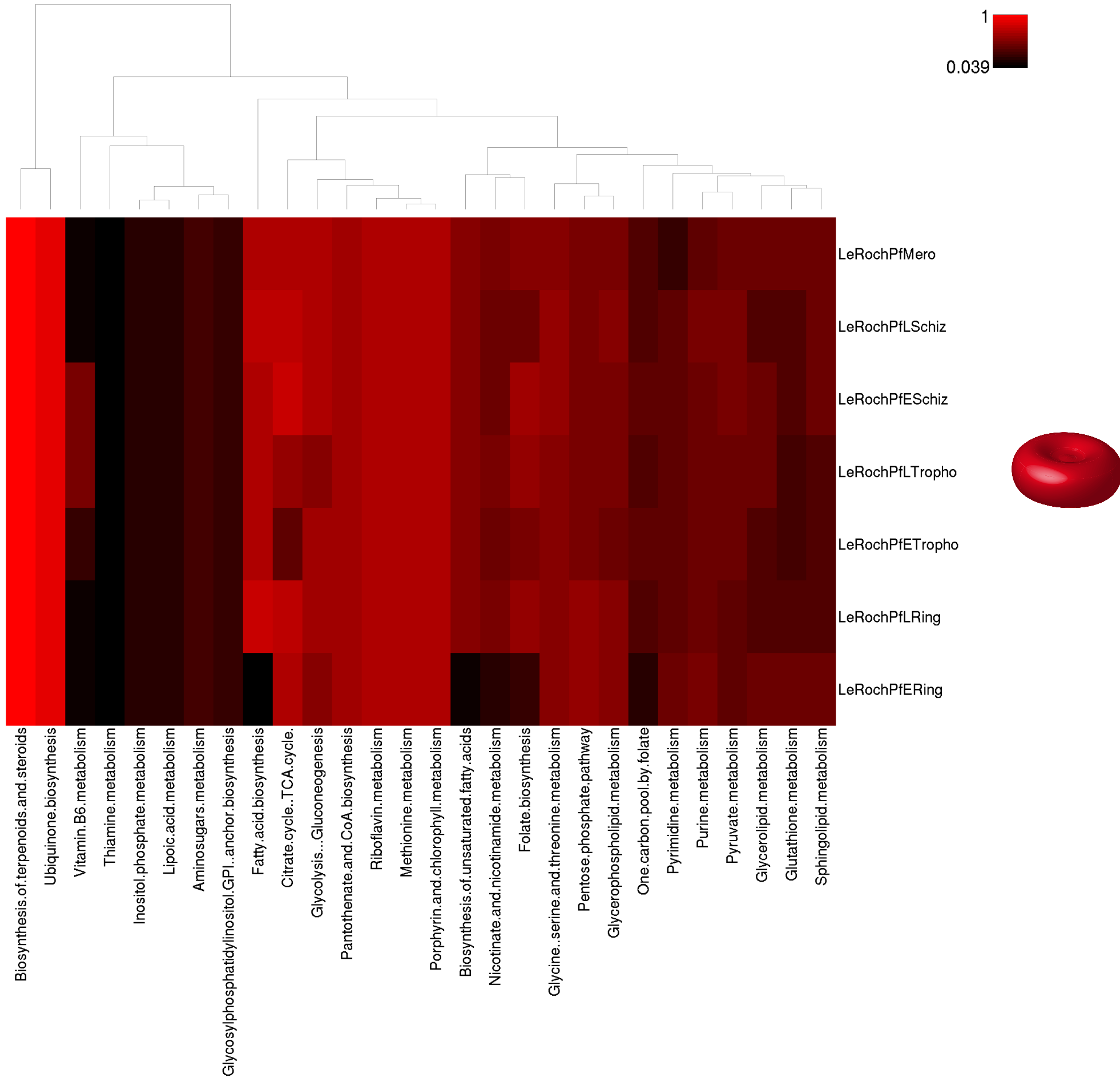

Supplement: Additional file 15 — Predicted metabolic fluxes consistent with Le Roch gene expression data mapped onto metabolic pathways. Same as Additional file 14 but fluxes were calculated using Le Roch gene expression data. [file 1752-0509-4-120-S15.PDF]

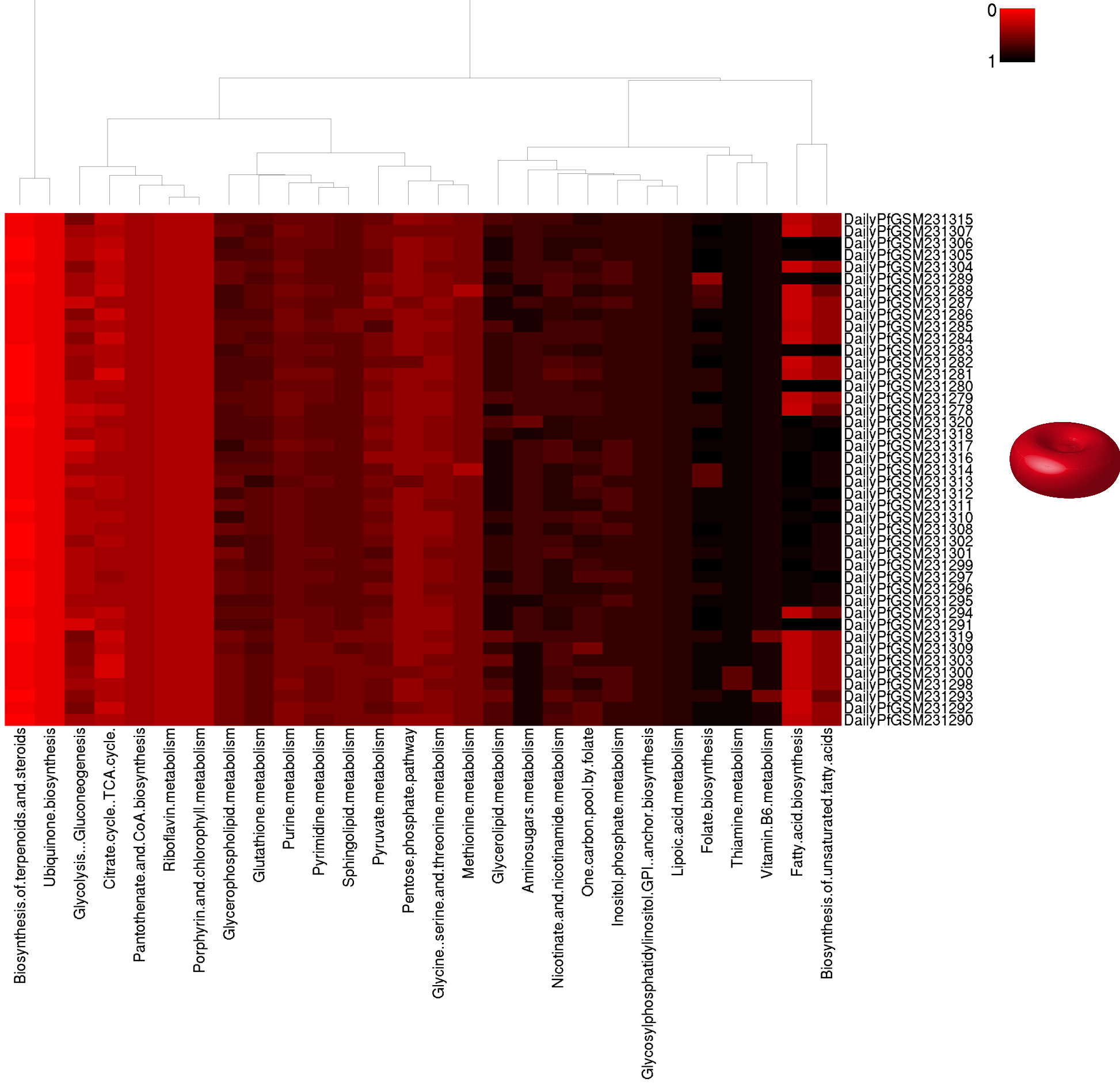

Supplement: Additional file 16 — Predicted metabolic fluxes consistent with Daily gene expression data mapped onto metabolic pathways. Same as Additional file 14 but fluxes were calculated using Daily gene expression data. [file 1752-0509-4-120-S16.PDF]

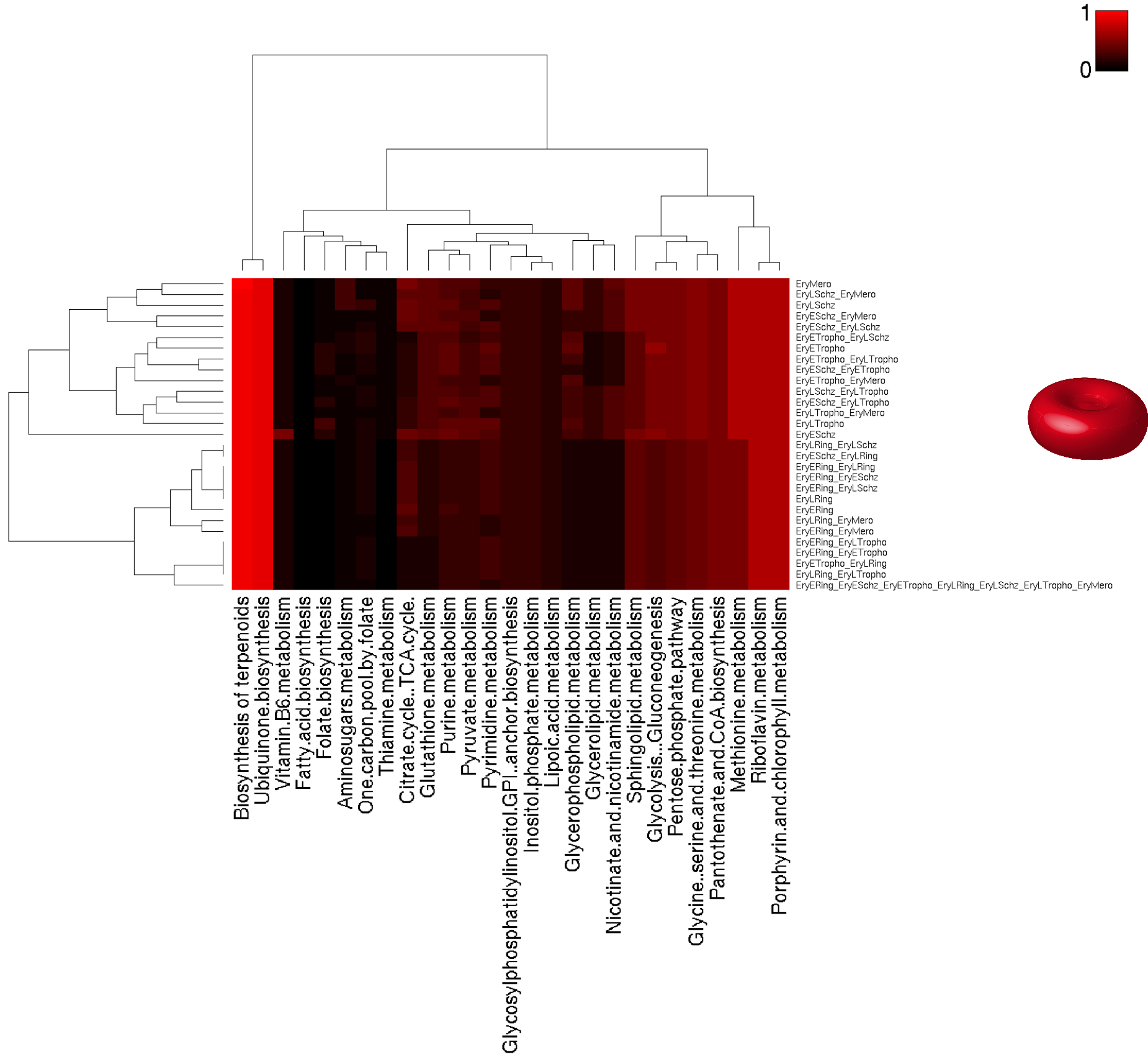

Supplement: Additional file 17 — Overview of pathway specific consensus reactions for different time points of intraerythrocytic cycle. Flux distributions have been predicted with our improved flux balance approach (see Figure 1) for each time point of the intraerythrocytic developmental cycle for which a gene expression profile exists. Simulations were conducted on the basis of the combined metabolic network of parasite and host and additional constraints reflecting knowledge about the blood stage. Furthermore, the expression status of genes during preceding time points was considered for the flux calculations. Consensus reactions, which are reactions that are predicted to be active for all gene expression samples covering the same stage, were determined. These reactions are more likely to actually occur during a certain stage, since they are derived from different data samples. In order to identify consensus reactions we grouped all calculated flux profiles corresponding to the blood stage into seven sets (eRing, lRing, eTropho, lTropho, eSchiz, lSchiz, Mero; see Additional file 3) with respect to represented stages and determined those reactions that carry a non-zero flux in all flux profiles of the same set. For each blood stage and each metabolic pathway the fraction of consensus reactions per total number of pathway reactions was computed to uncover the distribution of consensus reactions among pathways. To get an impression of how many consensus reactions are shared between the different stages, this fraction was also computed for those consensus reactions that two sets have in common. The darker the color of a matrix entry the lower is the percentage of consensus reactions. [file 1752-0509-4-120-S17.PDF]
