# Supplementary material for: Antimalarial drug targets in Plasmodium falciparum predicted by stage-specific metabolic network analysis
Source: BMC Syst Biol. 2010 Aug 31;4:120. doi: 10.1186/1752-0509-4-120 (PMC2941759; doi:10.1186/1752-0509-4-120)
Supplement: Additional file 11 — Predicted host parasite metabolite exchanges. Flux distributions have been predicted with our flux balance approach (see Figure 1) for each time point of the parasite's life cycle for which a gene expression profile exists. Simulations were conducted considering only the metabolic network of the parasite without any further constraints reflecting the parasite's environment and without considering the expression status of genes during preceding time points. Resulting metabolite exchanges between host and parasite are depicted in this figure. Red matrix entries represent metabolites that are predicted to be imported into the parasite while green matrix entries represent metabolites secreted into the host compartment. [file 1752-0509-4-120-S11.PDF]

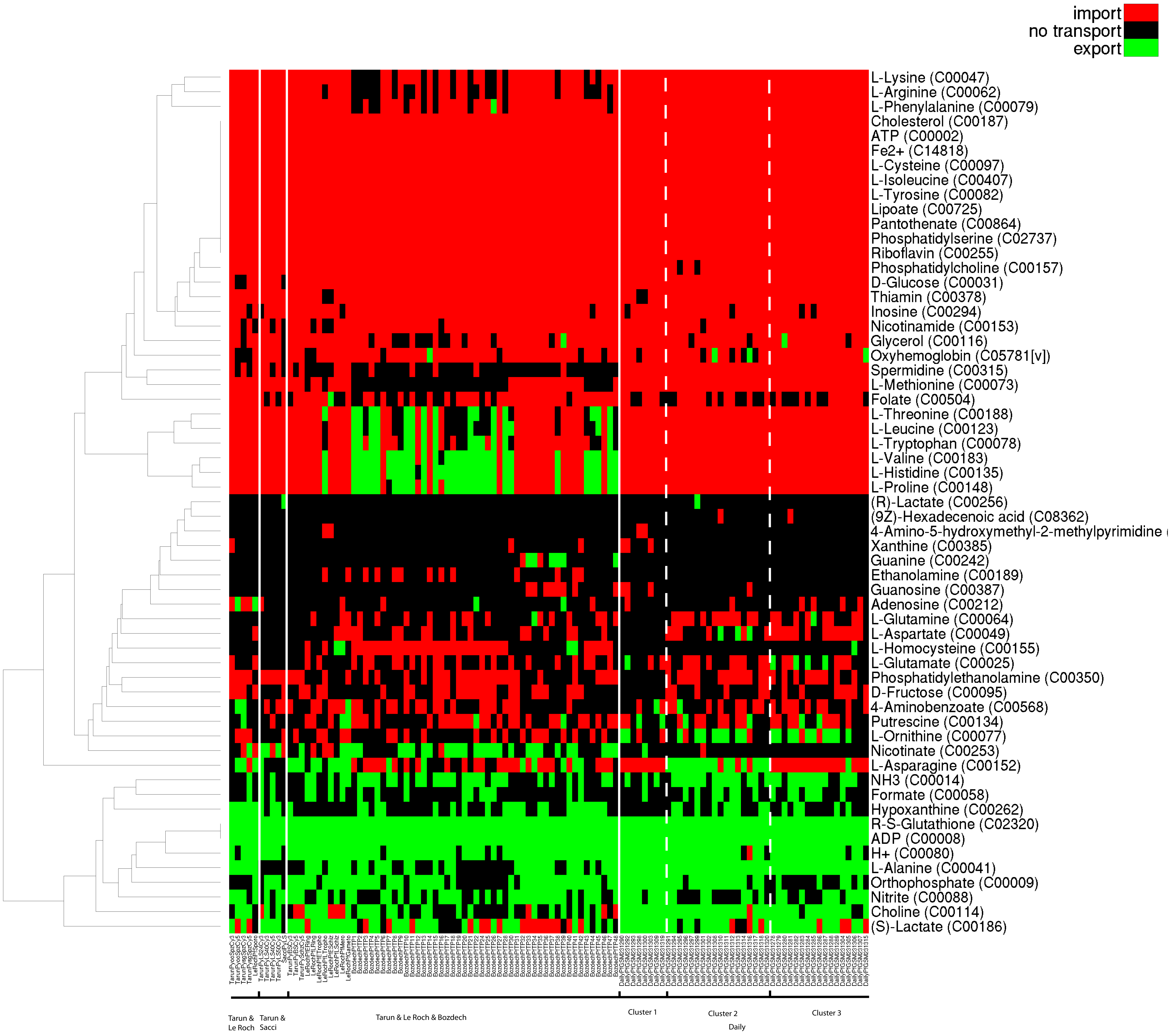
import
no transport
export
L-Lysine (C00047)
L-Arginine (C00062)
L-Phenylalanine (C00079)
Cholesterol (C00187)
ATP (C00002)
Fe2+ (C14818)
L-Cysteine (C00097)
L-Isoleucine (C00407)
L-Tyrosine (C00082)
Lipoate (C00725)
Pantothenate (C00864)
Phosphatidylserine (C02737)
Riboflavin (C00255)
Phosphatidylcholine (C00157)
D-Glucose (C00031)
Thiamin (C00378)
Inosine (C00294)
Nicotinamide (C00153)
Glycerol (C00116)
Oxyhemoglobin (C05781[v])
Spermidine (C00315)
L-Methionine (C00073)
Folate (C00504)
L-Threonine (C00188)
L-Leucine (C00123)
L-Tryptophan (C00078)
L-Valine (C00183)
L-Histidine (C00135)
L-Proline (C00148)
(R)-Lactate (C00256)
(9Z)-Hexadecenoic acid (C08362)
4-Amino-5-hydroxymethyl-2-methylpyrimidine (
Xanthine (C00385)
Guanine (C00242)
Ethanolamine (C00189)
Guanosine (C00387)
Adenosine (C00212)
L-Glutamine (C00064)
L-Aspartate (C00049)
L-Homocysteine (C00155)
L-Glutamate (C00025)
Phosphatidylethanolamine (C00350)
D-Fructose (C00095)
4-Aminobenzoate (C00568)
Putrescine (C00134)
L-Ornithine (C00077)
Nicotinate (C00253)
L-Asparagine (C00152)
NH3 (C00014)
Formate (C00058)
Hypoxanthine (C00262)
R-S-Glutathione (C02320)
ADP (C00008)
H+ (C00080)
L-Alanine (C00041)
Orthophosphate (C00009)
Nitrite (C00088)
Choline (C00114)
(S)-Lactate (C00186)
Tarun & Le Roch
Tarun & Sacci
Tarun & Le Roch & Bozdech
Cluster 1
Cluster 2
Cluster 3
Daily
